# Whole Genome Interpretation for a Family of Five

**DOI:** 10.3389/fgene.2021.535123

**Published:** 2021-03-08

**Authors:** Manuel Corpas, Karyn Megy, Vanisha Mistry, Antonio Metastasio, Edmund Lehmann

**Affiliations:** ^1^Cambridge Precision Medicine Limited, ideaSpace, University of Cambridge Biomedical Innovation Hub, Cambridge, United Kingdom; ^2^Institute of Continuing Education Madingley Hall Madingley, University of Cambridge, Cambridge, United Kingdom; ^3^Facultad de Ciencias de la Salud, Universidad Internacional de La Rioja, Madrid, Spain; ^4^Department of Haematology, University of Cambridge & National Health Service (NHS) Blood and Transplant, Cambridge, United Kingdom; ^5^Fabric Genomics, Oakland, CA, United States; ^6^Camden and Islington NHS Foundation Trust, London, United Kingdom

**Keywords:** whole genome sequencing, personal genomics, interpretation, precision medicine, genetic risk score, pharmacogenomics, nutrigenomics

## Abstract

Although best practices have emerged on how to analyse and interpret personal genomes, the utility of whole genome screening remains underdeveloped. A large amount of information can be gathered from various types of analyses via whole genome sequencing including pathogenicity screening, genetic risk scoring, fitness, nutrition, and pharmacogenomic analysis. We recognize different levels of confidence when assessing the validity of genetic markers and apply rigorous standards for evaluation of phenotype associations. We illustrate the application of this approach on a family of five. By applying analyses of whole genomes from different methodological perspectives, we are able to build a more comprehensive picture to assist decision making in preventative healthcare and well-being management. Our interpretation and reporting outputs provide input for a clinician to develop a healthcare plan for the individual, based on genetic and other healthcare data.

## Introduction

A great deal of literature has been generated over the past decade defining best practices for clinical interpretation of personal genomes (Nykamp et al., 2017; Biesecker et al., 2018; Brandt et al., 2019; Machini et al., 2019). Some additional approaches involve the simultaneous analysis of parents and child, for example in the case of pediatric diagnosis for children with rare diseases (Wright et al., 2018). Other studies have used family genomes to assign the precise chromosomal position of variants (Roach et al., 2011). To our knowledge, however, the use of genome analysis for screening and disease prevention remains underdeveloped. To address this shortcoming, our current study sheds light on two areas. First, we provide a comprehensive whole genome analysis of pathogenicity screening, genetic risk, pharmacogenomic, fitness, and nutrition trait analysis. Second, we discuss the joint interpretation of these results within the perspective of a family of five for whom we have deep phenotypic knowledge, allowing us to find “true positive” predictions based on the family observations.

In the past, we have performed assessment of personal genome analysis for the same set of family individuals using direct to consumer data and crowdsourcing methods (Glusman et al., 2012; Corpas et al., 2015). We were limited by the amount of data available at the time (DNA chip or exome) as well as a lack of reference data sources and analysis platforms to help with the interpretation that have appeared in recent years [e.g., gnomAD (Karczewski et al., 2019), ClinVar (Landrum et al., 2014)]. In this new iteration, we perform whole genome sequencing analysis for the same family of five and expand from our previous research to encompass a more comprehensive set of analyses and individual genomic data following published standard practice for interpretation of results as much as possible. Following standard practice is not always possible given that authoritative guidelines for interpretation of variants (Richards et al., 2015) are mostly applied to pathogenicity screening, rather than preventative healthcare using personal genomes. We were indeed able to perform a pathogenicity screening for the five members of the family quintet. For four genomes we also report genetic risk scores for 49 phenotypes using published Genome Wide Association Study (GWAS) markers (see Supplementary Table 1). We make a distinction between our genetic marker score notation and polygenic risk scores in the literature (Khera et al., 2018; Georgi et al., 2019; Meisner et al., 2019; Palmer, 2019; Torkamani and Topol, 2019) as we only use markers reported above a certain threshold of probability (as defined by GWAS studies).

To date, genome analysis of pathogenicity screening, genetic risk scoring for cardiovascular disease and some pharmacogenomics characterization has been performed by the MedSeq project for 100 individuals (Machini et al., 2019). Compared to this study, we offer novel perspectives on several fronts: (1) Our genetic risk analysis encompasses mental, metabolic, and autoimmune diseases, in addition to only cardiovascular being done in the MedSeq Project. (2) We include a systematic curation of known nutrition and fitness markers following newly developed guidelines to evaluate the scientific validity of gene x lifestyle interventions (Grimaldi et al., 2017). (3) Our deep phenotype and clinical knowledge of analyzed participants, helps us interpret and report results in a familial context within a wellness and prevention point of view. In addition, this work provides a proof-of-principle approach about an application of genetic risk scores within a family-oriented preventative healthcare and well-being case, recognizing that we are studying only one family and therefore this represents only an illustration of our proposed methodology for comprehensive whole genome analysis. Whenever possible, we use established guidelines from the American College for Medical Genetics and Genomics (ACMG), Food and Drug Administration (FDA), the Clinical Pharmacogenetics Implementation Consortium (CPIC), and other specialized organizations. We also discuss how different whole genome analysis methods can be integrated into more actionable outcomes for the individual and his or her relatives.

## Methods

### Ethical Framework

This project builds on prior work (Glusman et al., 2012; Corpas et al., 2015). We started as an open source project in 2010 using the data available from direct to consumer providers. As the project evolved and exome sequencing was performed, a consent form was created and signed for the collection of samples, analysis, and publishing of results. This form identified participants as voluntary donors of their genetic data to the public domain and educated participants, making them aware of the potential discomforts and risks that doing this research might bring.

Here we base our analysis on the whole genome rather than the exome. To facilitate this work, further collection of samples has been performed in order to sequence and analyse whole personal genomes for this family. All participants underwent a new consent process and signed a consent form accepting the terms and conditions of this work as well as the potential consequences of performing such analysis. When developing the consent framework, we drew on the Personal Genome Project UK (PGP-UK Consortium, 2018) as an example of a rigorous approach to informed consent. As a result, the consent process developed for this work included the following elements: (a) participants underwent extensive training on the risks of genetic analysis including the risks of publishing personal genetic data; (b) participants completed an exam to demonstrate their comprehension of the risks and protocols associated with participating in genetic analysis which may be published and (c) participants were judged truly capable of giving informed consent. Consent forms were signed by all family individuals or their next to kin (in the case of a deceased member). This ethical framework has been independently assessed and approved by the Ethics Committee of Universidad Internacional de La Rioja (code PI:029/2020).

### Family Dataset

**Figure 1:**
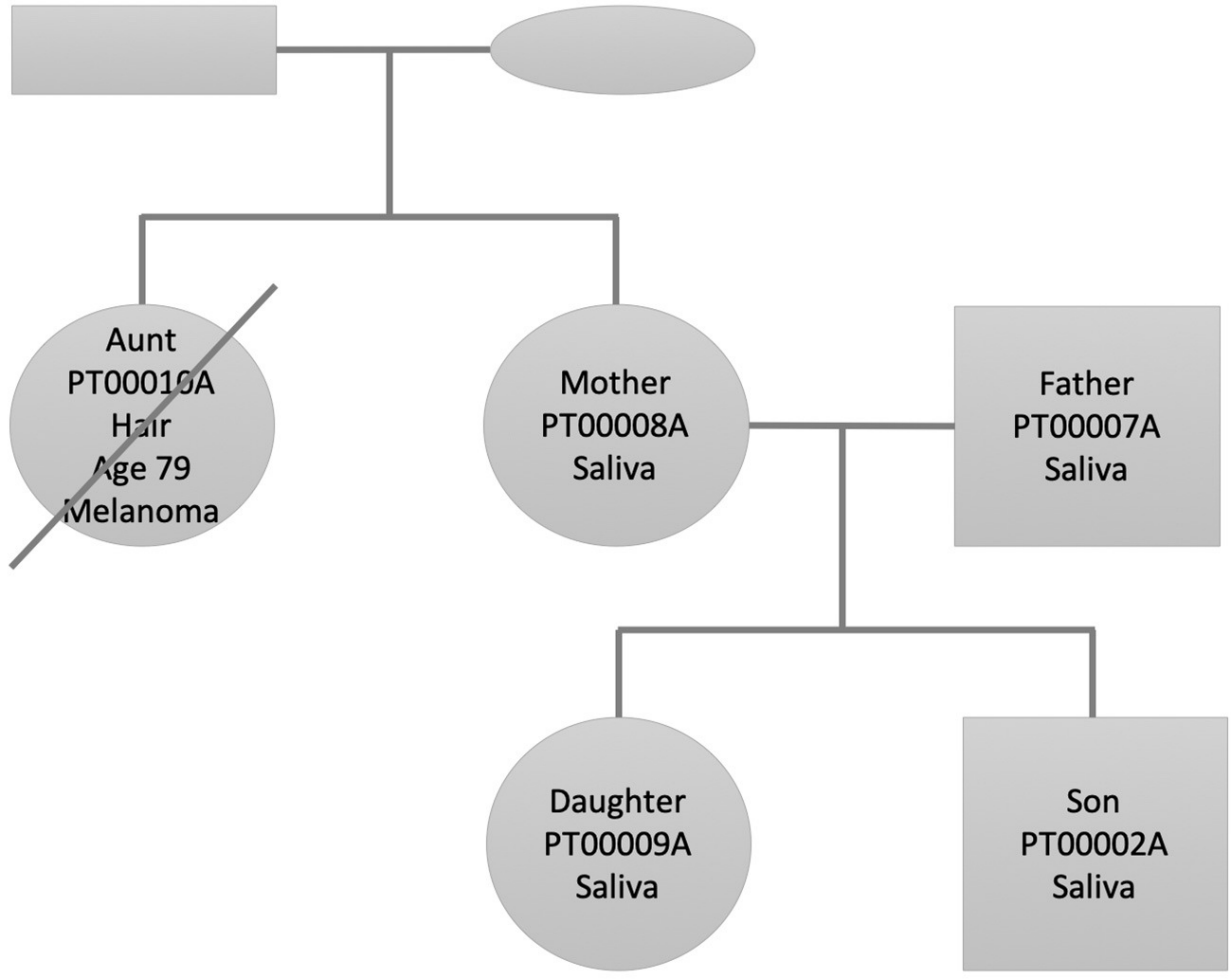
Family pedigree showing the relationship, gender (square: male, circle: female), and sample used for whole genome sequencing (saliva/hair). The crossed circle indicates a deceased individual.

We selected this family dataset for two reasons: (1) We have performed and published in the past decade two studies describing state of the art personal genomics analysis for a family of related individuals using array chip data and Illumina exome data (Glusman et al., 2012; Corpas et al., 2015). (2) The accumulated genetic studies and follow up of the disease and lifestyle history of the family through their continuous research have afforded us a deep knowledge of their phenotypes and disease history. Figure 1 shows the family pedigree. In it we have individuals PT00010A (Aunt), who is the sister of PT00008A (Mother). PT00007A (Father) is Mother's spouse and both have two children (PT00009A and PT00002A; Daughter and Son). From here onwards, and for simplicity, we refer to family members as (Aunt, Father, Mother, Daughter, Son). All individuals of the family except Aunt had their DNA sequenced from saliva, whereas Aunt's DNA was sequenced from hair (see Supplementary Materials for details). This is because at the time of sample collection Aunt was already deceased (see next section for phenotypic details). Thirty-six hairs were retrieved from a personal comb only she used and her DNA extracted from hair roots using a different protocol described in the Supplementary Materials.

When we analyzed the variant output of all samples, we benchmarked against Fabric Genomics Clinical Grade Scoring Rules (http://help.fabricgenomics.com/hc/en-us/articles/206433937-Appendix-4-Clinical-Grade-Scoring-Rules; accessed 7/January/2020), where Clinical Grade is a measure of a variant file's overall quality and fitness for clinical interpretation. The hair sample failed the criteria for clinical-grade coverage, genotype quality, homozygous/heterozygous ratio, and transition/transversion ratio (Table 1).

**Table 1:** Statistics for clinical grade measures of the quality of the variant file.

**Sample ID**	**Coverage**	**Genotype quality**	**Homozygous/heterozygous ratio**	**Transition/transversion ratio**
PT00002A (Son)	43.0*	94.3	0.51*	2.81*
PT00007A (Father)	25.0	95.9*	0.51*	2.81*
PT00008A (Mother)	24.0	95.7*	0.51*	2.79*
PT00009A (Daughter)	29.0	97.8*	0.48	2.79*
PT00010A (Aunt)	2.0	4.7	0.11	1.06

Star-marked values (*) indicates the quality is of clinical standards and no-star values that it is below clinical standards (see Fabric Genomics Clinical Grade Scoring Rules [http://help.fabricgenomics.com/hc/en-us/articles/206433937-Appendix-4-Clinical-Grade-Scoring-Rules]). Coverage in values with a star indicates that the median coverage of coding variants exceeds 40. Genotype quality with a starred value: more than 95% of the coding variants have a quality above 40. Starred homozygous/heterozygous ratio: the ratio for the coding variants is between 0.5 and 0.61. Starred transition/transversion ratio: The ratio for the coding variants is between 2.71 and 3.08. None of the quality measures for clinical grade sampling was met by Aunt, whereas clinical grade quality measures are reached by other individuals.

We performed a further analysis of quality of variants by counting those that pass the default standard filters of quality for interpretation given our analysis software (Table 2; see Supplementary Materials). For Aunt, we eliminated all variants below the threshold of QUAL < 20. The performance of the variant count and the level of coverage was sufficient to include Aunt in pathogenicity screening, but not sufficient for participation in the rest of the analysis.

**Table 2:** The total number of variants for all saliva samples and the total number of coding variants for each family member.

**Sample ID**	**Total number of variants**	**Total number of coding variants**
PT00002A (Son)	4,956,742	27,286
PT00007A (Father)	4,650,536	27,504
PT00008A (Mather)	4,695,886	27,329
PT00009A (Daughter)	4,812,818	27,400
PT00010A (Aunt)	970,018	16,182

### Family History of Lifestyle and Disease

We conducted research into the family disease and lifestyle history. This research consisted of face-to-face interviews with all family members, during which they were asked about past illnesses, hospitalizations, reasons of death for past relatives and any ongoing condition that they think might related to the phenotypes and traits we analyse in this study. At the time of our last interview (October 2020), Mother and Father are in their mid-eighties, a similar age Aunt would be, had she not passed due to metastasised melanoma at age 79. Daughter is in her late fifties and Son mid-forties. All members of the family have been diagnosed obese or overweight at some point in their adulthood years. Childhood obesity was present in both Son and Daughter. Mother had a benign breast tumor removed in her early forties. She has also suffered from a history of low blood pressure and was diagnosed with chronic inflammation of her colon in her sixties, suffering from lower abdominal pain ever since. Father has a history of high blood pressure and heart problems. He has recently been diagnosed with atrial fibrillation. He displays difficulty breathing at moderate exertion levels and has been taking anticoagulants to prevent thromboembolism as a consequence of his atrial fibrillation, with some episodes of adverse drug reactions to warfarin. He is suspected to be lactose intolerant. In addition to her metastasised melanoma, before Aunt's passing she suffered from several episodes of venous thromboembolism, treated with anticoagulants (warfarin). There is no history in the family of diabetes or Parkinson's disease, although the father of both Mother and Aunt was diagnosed with Alzheimer's disease in his mid-eighties. Apart from Aunt's melanoma, there is no history of any other malignancy known to the family, no major mental health episodes or alcohol dependence diagnosed to date. All family members except Mother reported being light smokers for a period of their lives, all having quit more than a decade ago except daughter who still smokes several cigarettes a day.

### Pathogenicity Screening

All single nucleotide variants and indels were filtered according to three different gene panels: (1) genes present in the OMIM morbid list (Amberger et al., 2015), (2) ACMG 59 genes (Kalia et al., 2017), and (3) a Hereditary Cancer panel of 52 genes (https://info.fabricgenomics.com/ace; accessed 10/February/2020). All three panels required pathogenic or likely pathogenic alleles matching ClinVar (Landrum et al., 2014) evidence. The variant prioritization was based on their ClinVar evidence, their frequency in gnomAD (Karczewski et al., 2019), the 1000 Genomes Project (The 1000 Genomes Project Consortium, 2015) and their predicted variant effect (i.e., loss of function, non-synonymous or other). Our selection of frequency threshold is based on the gnomAD database (https://gnomad.broadinstitute.org/faq) criteria of common variant sites, defined as frequency >0.01. For each of the variants that passed the filtering, we classified them following the guidelines proposed by the ACMG (Richards et al., 2015) into 5 categories; from most to least pathogenic these categories are: pathogenic, likely pathogenic, uncertain significance, likely benign, benign. Relevant scientific literature as well as a number of algorithms were also used to assess each prioritized variant [i.e., OMICIA (Coonrod et al., 2013), VAAST (Hu et al., 2013), VVP (Flygare et al., 2018), and CADD (Rentzsch et al., 2019)].

### Genetic Risk Scores

Genetic risk scores, also called genetic predisposition scores, aim to quantify the cumulative effects of a number of variants affecting multiple genes, which may individually confer only small risk susceptibility. Genetic risk scores are not diagnostic, as a high-risk score does not necessarily mean that a person will develop a condition, and a low score does not mean that they will not develop it. Nevertheless, genetic risk scores may be pointers for further exploration when looking for potential preventative interventions, particularly for multigenic conditions like diabetes type 2, hypertension or many mental illnesses. They can be useful when other independent sources of risk information are also concordant [e.g., genotype/phenotype additional knowledge (Fahed et al., 2020), family history, imaging data]. A database of 4,688 published GWAS SNPs was generated encompassing 49 common diseases (we call these common diseases “phenotypes” from now onwards; Supplementary Table 1), their risk alleles and weighted contributions (odds ratio or beta scores). These phenotypes were selected according to GWAS Catalog criteria (https://www.ebi.ac.uk/gwas) as having studies including a primary GWAS analysis, defined as array-based genotyping and analysis of 100,000+ pre-QC SNPs selected to tag variation across the genome and without regard to gene content. Individual SNP-trait associations were collected with a statistical significance (SNP-trait *p*-value <1.0 x 10^−5^) in the overall (initial GWAS and replication) population. To create genetic risk scores, each collected SNP marker was required to possess (a) the risk allele and (b) the measurement or effect size that this risk allele confers to the individual that carries this mutation. A genetic risk score was calculated as the sum of the weights of all the phenotype's risk alleles observed in the individual divided by the total number of alleles reported for that phenotype. We used the final (Phase 3) dataset of the 1000 Genomes Project containing data for 2,504 individuals from 26 populations to calculate their genetic risk scores for each of the 49 phenotypes. The 1000 Genomes Project individuals became our background distribution of genetic risks against which to measure how far from the mean each of the family participant lies. We required that the identified GWAS SNPs are also present in the 1000 Genomes Project since individuals from the 1000 Genomes Project were used as a background population to which compare the participant's score. In order to control for potential differences in results due to the ethnic diversity of the background population, we also performed the analysis using a background population of only the 503 European (CEU) participants in the 1000 Genomes Project, given that all family members are of European origin.

We plotted the genetic risk score of each family member to establish whether he or she lies on the higher tail of the distribution of scores in relation to the calculated risk scores of 1000 Genomes Project individuals. In order to evaluate whether a member of the family had a reportable genetic risk, we applied a two standard deviations (2SD) threshold from the mean genetic risk score of the background 1000 Genomes individual distribution for a particular phenotype (equivalent to the top 5 percentile normal distribution of a predicted risk). We use a threshold of 2SD to give confidence that results are not attributable to chance. Furthermore, scores from both 1000 Genome individuals and family members are calculated independently. Multiple testing correction is not performed since the objective here is to identify family members in the extreme risk tail of the 1000 Genomes background distribution of calculated scores. For completeness, we also noted those phenotypes for which a greater than one standard deviation (1SD) from the mean background genetic risk is reached by the tested family individual.

### Pharmacogenomics

We analyzed three well-known genes influencing pharmacogenomic responses, all of them forming part of the Cytochrome P450 family: *CYP2D6, CYP2C9, CYP2C19*. In order to extract relevant pharmacogenomic data, we rely on Food and Drug Administration (FDA) and European Medicines Agency (EMA) guidance sourced via the PharmGKB database (https://www.pharmgkb.org). We also take guidance from the Clinical Pharmacogenetics Implementation Consortium (CPIC; https://cpicpgx.org), the Association for Molecular Pathology and College of American Pathologists (https://www.amp.org), and the American College of Medical Genetics and Genomics (https://www.acmg.net). In order to perform the genotyping of pharmacological genes we allow the extraction of non-variant positions if required.

The testing of specific positions within a gene provides an accurate representation of the metaboliser status of an individual for that particular gene. For instance, if we were to assume that an individual has a nucleotide change 1846G>A in *CYP2D6*, this polymorphism is determinant for allele ^*^4. If there are no other variants, it is presumed that the other allele this person harbors is a wild haplotype, being denoted as ^*^4/^*^1 [see (Nofziger et al., 2020) for more detail]. Once both haplotypes for an individual are identified, a lookup table is referenced where the pharmacological effect of the observed haplotypes are indexed.

Pharmacological analysis also depends on whether analyzed genes have their copy number altered. We performed a consensus-based algorithm prediction using the short-read sequencing data for Father, Mother, Daughter and Son (Supplementary Materials). This analysis did not yield significant evidence for presence of copy number alterations in all Cytochrome P450 genes analyzed here.

### Fitness and Nutrition

Besides pathogenicity screening, genetic risk scoring, and pharmacogenomics, there is further useful information that can be extracted from whole genomes using genotyping. In particular, we identify two areas of interest that provide further information about a person's genetic load: fitness and nutrition. We recognize that these areas of genetic analysis are less developed than pathogenicity screening, and so we add rigor to the analysis by first evaluating the quality of supporting evidence before testing for the presence of variants in the family. To evaluate the scientific validity and evidence for genotype-based dietary or fitness advice, we first performed a literature search to identify an initial list of potential genetic markers, and then adopted the proposed recommendations of Grimaldi et al. (2017) for specific gene x interactions and their relation to a health outcome. This framework allows us to establish levels of confidence for each of the SNPs or groups of SNPs we analyse for fitness and nutrition, according to a set of peer-reviewed guidelines. These guidelines differentiate four levels of scientific evidence assessment:

- “Convincing”: gene x interaction is based on at least 3 studies with high subject numbers, showing the relation and mechanistic knowledge.
- “Probable” is based on several studies showing the relation and/or some mechanistic understanding.
- “Possible”: based on a few studies showing the relation.
- “Not demonstrated” is any level of evidence below the established above.

The levels of assessment above rely on the following criteria:

- “Study quality rating”: either A, B, C or D, based on whether a study is (a) interventional or observational; (b) prospective or retrospective, (c) whether it is randomized, placebo controlled and blinded; (d) the number of subjects with effect alleles (where possible); (e) the effect magnitude; (f) *P*-values, false discovery rate and multiple testing and; (g) replications in other populations and meta-analyses.
- “Type of gene x interaction”: direct phenotype, intermediate phenotype, or indirect phenotype.
- “Nature of genetic variant”: causal, in linkage disequilibrium with functional variant or associated but unknown function.
- “Biological plausibility,” rated as high, medium, low, or unknown, based on our critical assessment of current understanding of the physiological effect of identified SNPs.

Our initial selection and classification of genotyping markers for fitness and nutrition are shown in Tables 3, 4. We then assess each marker according to the above criteria of scientific evidence, carrying forwards for analysis in the family participants those classified as convincing (fitness *n* = 2; nutrition *n* = 13) and probable (fitness *n* = 5; nutrition *n* = 1).

**Table 3:** Summary of fitness trait analysis candidates assessed according to the scientific validity score as proposed by Grimaldi et al. (2017).

**Category**	**Trait**	**RSID**	**Gene**	**Study quality rating**	**Type of gene x trait interaction**	**Nature of genetic variant**	**Biological plausibility**	**Number of independent studies**	**Total number subjects studied**	**Knowledge of biological mechanism involved**	**Scientific validity score**	**References**
Fitness	VO_2_max	21 SNPs	Multiple	A	Direct phenotype	Causal	High	35	>1000	Medium	Convincing	Rice et al., 2012; Ghosh et al., 2013; Williams et al., 2017
Fitness	Muscle performance	rs1815739	ACTN3	A	Indirect phenotype	Causal	High	24	>1000	High	Convincing	Kikuchi et al., 2014, 2015, 2017b; Schadock et al., 2015; Yvert et al., 2015, 2016; Baumert et al., 2016; Itaka et al., 2016; Min et al., 2016; Del Coso et al., 2017, 2019a,b; Galeandro et al., 2017; Houweling et al., 2018; Zhang et al., 2019; Baltazar-Martins et al., 2020; Calvano Küchler et al., 2020; Murtagh et al., 2020; Płóciennik et al., 2020
Fitness	Caffeine sensitivity/Increased exercise performance with caffeine	rs762551	CYP1A2	C	Direct phenotype	Causal	High	7	250	High	Probable	Pataky et al., 2016; Salinero et al., 2017; Guest et al., 2018; Puente et al., 2018; Carswell et al., 2020; Grgic et al., 2020; Muñoz et al., 2020
Fitness	Endurance	rs4253778	PPARA	B	Indirect phenotype	Causal	Medium	6	3267	High	Probable	Ahmetov et al., 2009; Ahmetov and Fedotovskaya, 2015; Lopez-Leon et al., 2016; Petr et al., 2019; Johansen et al., 2020; Murtagh et al., 2020
Fitness	Lactate blood levels	rs1049434	MCT1	B	Direct phenotype	Causal	High	4	2048	High	Probable	Cupeiro et al., 2012; Fedotovskaya et al., 2014; Ben-Zaken et al., 2015; Kikuchi et al., 2017a
Fitness	Osmotic balance by water support	rs1049305	AQP1	B	Indirect phenotype	Causal	High	3	2613	Medium	Probable	Saunders et al., 2015; Rivera and Fahey, 2019; Rivera et al., 2020
Fitness	Performance	rs12594956	NRF-2	C	Indirect phenotype	Causal	High	4	1598	Medium	Probable	He et al., 2007; Eynon et al., 2010, 2013; Peplonska et al., 2017
Fitness	Glucose transportation and lipid and glucose oxidation	rs8192678	PPARGC1A	C	Indirect phenotype	Causal	High	5	409	Medium	Possible	Petr et al., 2018
Fitness	Endurance	rs12722	COL5A1	C	Indirect phenotype	Causal	High	3	952	Medium	Possible	O'Connell et al., 2013; Bertuzzi et al., 2014; Murtagh et al., 2020
Fitness	Elite endurance	rs4994	ADRB3	D	Indirect phenotype	Causal	High	2	453	Low	Not demonstrated	Gómez-Gallego et al., 2010; Santiago et al., 2011

*A total of 10 fitness traits were identified for their gene x interaction assessment. We classified as “Convincing” those traits whose gene x interaction is based on at least 3 studies with high subject numbers, showing the relation and mechanistic knowledge. A trait classified as “Probable” is based on several studies showing the relation and/or some mechanistic understanding. A trait is deemed “Possible” if based on a few studies showing the relation. “Not demonstrated” are those traits for which any level of evidence is below the established criteria above*.

**Table 4:** Summary of nutrition trait analysis candidates assessed according to the scientific validity score as proposed by Grimaldi et al. (2017).

**Category**	**Trait**	**RSID**	**Gene**	**Study quality rating**	**Type of gene x trait interaction**	**Nature of genetic variant**	**Biological plausibility**	**Number of independent studies**	**Total number subjects studied**	**Knowledge of biological mechanism involved**	**Scientific validity score**	**References**
Nutrition	Homocystine levels	rs1801133	MTHFR	A	Direct phenotype	Causal	High	70	>100000	High	Convincing	Boccia et al., 2008, 2009; Clarke et al., 2011; Liew and Gupta, 2015
Mental health / Nutrition	Alzheimer's	rs429358,rs7412	APOE	A	Direct phenotype	Causal	High	146	>100000	High	Convincing	Martins et al., 2006; Zhang et al., 2015; Rasmussen et al., 2018
Nutrition	Alcohol dependence	rs1229984	ADH1B	A	Direct phenotype	Causal	High	59	>100000	High	Convincing	Jorgenson et al., 2017; Katsarou et al., 2017; Masaoka et al., 2017; Wolf et al., 2017; Hubacek et al., 2018; Justice et al., 2018; Polimanti and Gelernter, 2018; Walters et al., 2018; Yokoyama et al., 2018, 2019, 2020a,b,c; Howe et al., 2019; Johnson et al., 2019; Lai et al., 2019; Sun et al., 2019; Szentkereszty-Kovács et al., 2019; Thompson et al., 2020
Nutrition	Greater total body adiposity	rs9939609	FTO	A	Direct phenotype	Causal	High	25	>100000	Medium	Convincing	Bollepalli et al., 2010; Dedoussis et al., 2011; Mangge et al., 2011; Dwivedi et al., 2012; Lauria et al., 2012; Meng et al., 2014; Zhang et al., 2014; Zhao et al., 2014a; Qi et al., 2015a; Quan et al., 2015; Duicu et al., 2016; García-Solís et al., 2016; Livingstone et al., 2016; Bordoni et al., 2017; Almeida et al., 2018; Ferreira Todendi et al., 2019; Ranzenhofer et al., 2019; Todendi et al., 2020
Nutrition	Vitamin D Metabolism	rs4588	GC	B	Direct phenotype	Causal	High	21	>100000	High	Convincing	Robien et al., 2013; Nissen et al., 2014, 2015; Pekkinen et al., 2014; Braithwaite et al., 2015; Madden et al., 2015; Touvier et al., 2015; Petersen et al., 2017; Yao et al., 2017; Chuaychoo et al., 2018; Karuwanarint et al., 2018; Al-Daghri et al., 2019; Bahrami et al., 2019; Enlund-Cerullo et al., 2019; Mehramiz et al., 2019; Rahimi et al., 2019; Zhou et al., 2019; Gibbs et al., 2020a,b
Nutrition	Vitamin B12 level	rs602662	FUT2	A	Direct phenotype	Causal	High	6	>9000	High	Convincing	Hazra et al., 2009; Tanaka et al., 2009; Tanwar et al., 2013; Allin et al., 2017; Nongmaithem et al., 2017; Zhao and Schooling, 2017
Nutrition	Vitamin C level	rs33972313	SLC23A1	A	Direct phenotype	Causal	High	12	>100000	High	Convincing	Timpson et al., 2010; Duell et al., 2013; Amir Shaghaghi et al., 2014; Kobylecki et al., 2015, 2018; Wade et al., 2015; Ravindran et al., 2019
Nutrition	Vitamin E level	rs964184	BUD13/ZNF259	B	Direct phenotype	Causal	High	4	>10000	High	Convincing	Major et al., 2011, 2012, 2014; Wang and Xu, 2019
Nutrition	Iron Overload /Hemochromatosis	rs1800562	HFE	B	Direct phenotype	Causal	High	4	>5000	High	Convincing	McLaren et al., 2011; Katsarou et al., 2016; Barton et al., 2018; Wilman et al., 2019
Nutrition	Saturated fat / risk of T2D	rs1137101	LEPR	C	Indirect phenotype	Causal	High	12	>10000	Medium	Convincing	Domínguez-Reyes et al., 2015; Yang et al., 2016
Nutrition	Polyunsaturated Fatty Acids	rs174547	FADS1	C	Direct phenotype	Causal	High	11	3713	Medium	Convincing	Huang et al., 2017; Ching et al., 2019; Wang et al., 2020
Nutrition	Lactose persistence	rs4988235	MCM6-LCT	A	Direct phenotype	Causal	High	>10	>100000	High	Convincing	Baffour-Awuah et al., 2015
Nutrition	Celiac disease	rs2187668	HLA-DQA1	A	Direct phenotype	Causal	High	Many	7249	High	Convincing	van Heel et al., 2007; Hunt et al., 2008
Nutrition	Saturated fat	rs5082	APOA2	B	Direct phenotype	Causal	High	3	2856	Medium	Probable	Yabuta et al., 2016; Moran et al., 2019; Amengual et al., 2020; Graßmann et al., 2020
Nutrition	Vitamin A level	rs6564851	BCO1	C	Direct phenotype	Causal	High	4	328	Medium	Possible	Delgado-Lista et al., 2007; Smith et al., 2013; Noorshahi et al., 2016
Nutrition	Total Carbohydrates	rs7578326	IRS1	B	Indirect phenotype	Causal	High	2	~2000	Medium	Possible	Zheng et al., 2013; Mahmutovic et al., 2019
Nutrition	Total Carbohydrates	rs2943641	IRS1	B	Indirect phenotype	Causal	High	2	~2000	Medium	Possible	Zheng et al., 2013; Mahmutovic et al., 2019
Nutrition	Sugar	rs7903146	TCF7L2	A	Indirect phenotype	Causal	High	2	26905	Medium	Possible	Hindy et al., 2012, 2016
Nutrition	Alcohol metabolism	rs698	ADH1C	C	Direct phenotype	Causal	High	Many	>100000	High	Possible	Bierut et al., 2010; Martínez et al., 2010; Olfson and Bierut, 2012; Kranzler et al., 2019
Nutrition	Sweet Foods / Sweet Tooth	rs838133	FGF21	A	Direct phenotype	Causal	Medium	1	6514	High	Not demonstrated	Søberg et al., 2017
Nutrition	Vitamin B6 level	rs4654748	ALPL	B	Direct phenotype	Causal	High	1	~3000	High	Not demonstrated	Tanaka et al., 2009
Nutrition	Total Carbohydrates	rs2241201	MMAB	C	Indirect phenotype	Causal	High	1	920	Low	Not demonstrated	Junyent et al., 2009
Nutrition	Fiber	rs4457053	ZBED3	B	Indirect phenotype	Causal	High	1	26905	Medium	Not demonstrated	Hindy et al., 2016
Nutrition	Fiber	rs10923931	NOTCH2	B	Indirect phenotype	Causal	High	1	26905	Medium	Not demonstrated	Hindy et al., 2016
Nutrition	Sugar	rs12255372	TCF7L2	B	Indirect phenotype	Causal	High	2	26979	Medium	Not demonstrated	Hindy et al., 2016; López-Ortiz et al., 2016
Nutrition	Total fat	rs324420	FAAH	C	Direct phenotype	Causal	High	5	>5000	Medium	Not demonstrated	Jensen et al., 2007; de Luis et al., 2011; Knoll et al., 2012; Balsevich et al., 2018; Doris et al., 2019
Nutrition	Saturated fat	rs12449157	GFOD2	D	Direct phenotype	Causal	High	1	41	Medium	Not demonstrated	Guevara-Cruz et al., 2013
Nutrition	Omega-3 Fatty Acids	rs17300539	ADIPOQ	C	Direct phenotype	Causal	High	1	310	Medium	Not demonstrated	Alsaleh et al., 2013
Nutrition	Saturated Fatty Acids	rs1800629	TNF	C	Indirect phenotype	Causal	High	2	472	Medium	Not demonstrated	Cormier et al., 2016; Oki et al., 2017
Nutrition	Protein	rs12785878	DHCR7	D	Indirect phenotype	Causal	High	1	732	Medium	Not demonstrated	Qi et al., 2015b
Nutrition	Calcium	rs2228570	VDR	C	Direct phenotype	Causal	High	3	>5000	Medium	Not demonstrated	Jenab et al., 2009; Slattery et al., 2010; Zhou et al., 2015
Nutrition	Zinc	rs73924411	SLC30A3	D	Direct phenotype	Causal	High	2	350	Low	Not demonstrated	da Rocha et al., 2014a,b

*A total of 32 nutrition traits were identified for their gene x interaction assessment. We classified as “Convincing” those traits whose gene x interaction is based on at least 3 studies with high subject numbers, showing the relation and mechanistic knowledge. A trait classified as “Probable” is based on several studies showing the relation and/or some mechanistic understanding. A trait is deemed “Possible” if based on a few studies showing the relation. “Not demonstrated” are those traits for which any level of evidence is below the established criteria above*.

Having made the selection of relevant SNPs according to the above framework, we proceed to analyse the family. The trait analysis is performed as follows. First, a list of all the positions of the SNPs to be tested is created. All those positions are queried in the VCF files for each of the family members and all observed alleles are then recorded. The observed alleles are then interpreted via lookup tables collected from the scientific literature.

An exception to the above approach concerns the phenotype susceptibility to VO_2_max trainability, where we use a specific study. To calculate the VO_2_Max trainability genetic score, we follow the methodology outlined in Bouchard et al. (2011), that identifies SNPs associated with improvements in VO_2_Max. This study provides a panel of 21 SNPs that accounted for 49% of the variance in VO_2_Max trainability.

## Results

### Pathogenicity Screening

**Figure 2:**
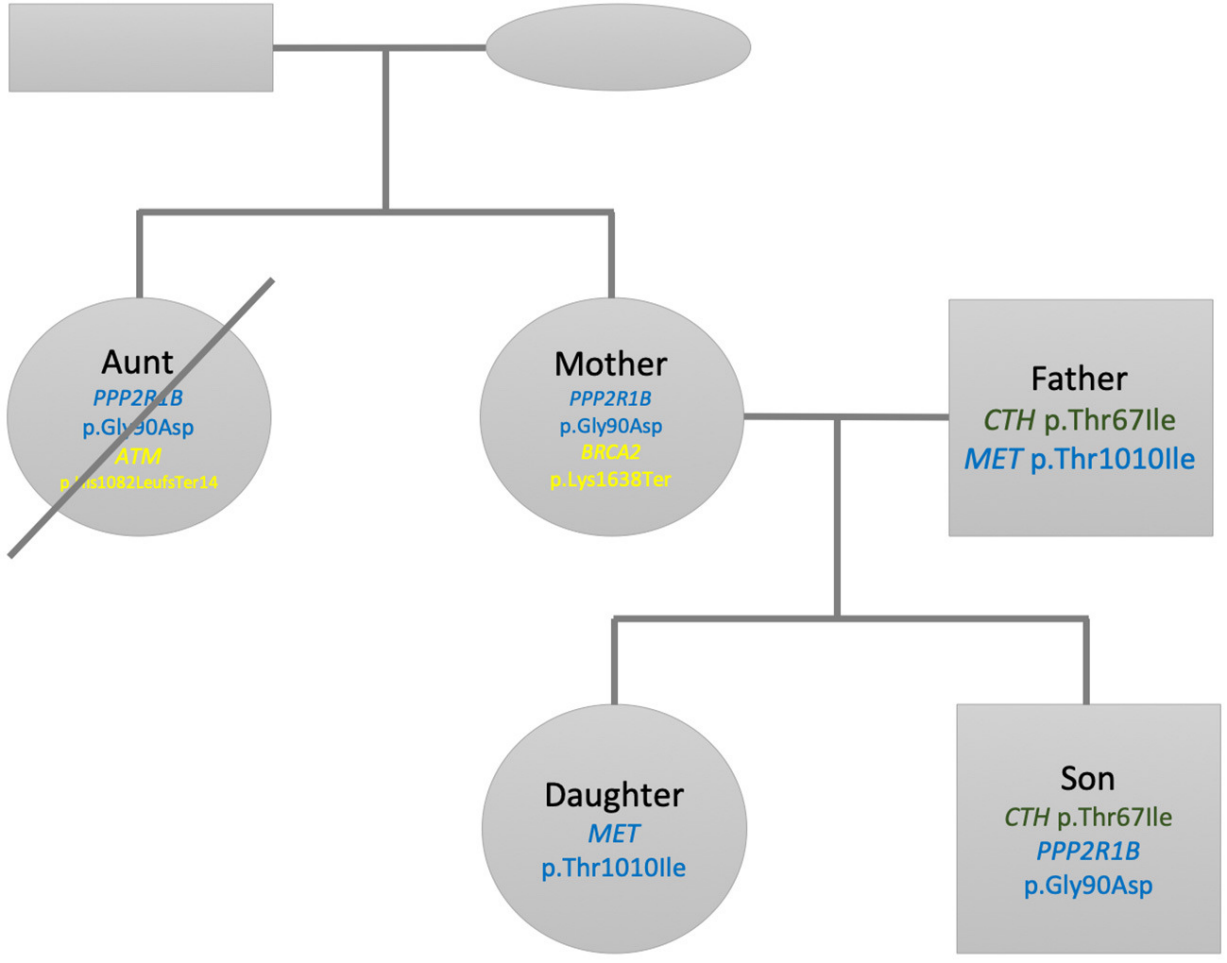
Family pedigree showing the relationship, gender (square: male, circle: female), and variants found listed within each individual. Green variants are inferred as benign, blue are variants of unknown significance and yellow pathogenic variants according to ACMG scoring. The crossed circle indicates a deceased individual.

Figure 2 shows a summary of the pedigree and filtered variants found listed within each individual. For Son, when searching for pathogenic or likely pathogenic mutations within a panel of 4,100 OMIM morbid genes, we found that only two mutations passed our prioritization and filtering criteria (see Methods section). No other mutations passed the threshold criteria within the ACMG59 and Hereditary Cancer panels. The first mutation is a heterozygous missense change of C → T; c.200C>T; p.Thr67Ile within the CTH gene. This change has been associated to cystathioninuria, a disorder observed in 1 out of 20,000 individuals (ORPHANET, Pavan et al., 2017). However, the ExAC (Lek et al., 2016) frequency in non-Finnish European is (~1 in 100), so much higher than the prevalence of the disorder. We also observe that this missense variant is not excessively constrained: its missense z-score is −0.127428 (excessively constrained genes are those with a missense z-score > 3.09, corresponding to a *p*-value < 0.001). Multiple lines of computational evidence suggest no impact on the gene. Our current assessment is that this variant is benign according to the ACMG scoring and inferred classification and is therefore not considered any further. The second mutation for Son corresponds to chr11:111764842 (rs1805076) producing C → T; c.269G>A; p.Gly90Asp in PPP2R1B. This gene encodes a regulatory subunit of protein phosphatase 2. Protein phosphatase 2 is one of the four major Ser/Thr phosphatases, and it is implicated in the negative control of cell growth and division. While ClinVar evidence suggests a matching allele to cause lung cancer, the computational and other sources of evidence are inconclusive, hence we infer this variant to be of uncertain significance (VUS).

For Father two variants pass the filters. The first variant is selected from the 4100 genes OMIM panel and corresponds to the heterozygous missense change of C → T; c.200C>T; p.Thr67Ile within the *CTH* gene, which is the same one Son has. We set the same classification as above and conclude it to be a benign variant as well. The second variant is located in the *MET* gene, part or our Hereditary Cancer panel, on chr7:116771936 (rs56391007) and produces C → T; c.3029C>T; p.Thr1010Ile. This gene encodes a member of the receptor tyrosine kinase family of proteins and the product of the proto-oncogene *MET*. The *MET* gene is associated with autosomal dominant hereditary papillary renal cell carcinoma (Takahashi et al., 2002). According to ORPHANET (Pavan et al., 2017), the prevalence of this cancer is less than 1 in 1,500,000, while the allele frequency of this variant is 1 in ~89 in non-Finnish European, higher than expected for the disorder. There are seven pathogenic and 15 likely pathogenic ClinVar missense variants in this gene while there are two benign and 25 likely benign ClinVar missense variants in this gene. Evidence thus indicates that missense variants are not a common mechanism of disease. In addition, there are multiple sources that point to this mutation being both pathogenic and likely benign. We conclude that this variant is of uncertain significance.

The first selected mutation for Mother comes from the OMIM disease gene panel and is the same as the one previously reported for Son corresponding to chr11:111764842 (rs1805076) producing C → T; c.269G>A; p.Gly90Asp in *PPP2R1B*. We apply the same criteria as above, inferring this variant effect being of uncertain significance. The second variant selected for interpretation in Mother corresponds to a heterozygous stop gained mutation in chr13:32339267 (rs886040553) producing the change A → T; c.4912A>T; p.Lys1638Ter in the *BRCA2* gene. The gene is included both in the ACMG 59 and our Hereditary Cancer gene panel. The impact of this mutation is stop-gained in a splice site. ClinVar evidence contains several entries in its classification history all matching the allele and all pathogenic. This variant is absent from gnomAD or the 1000 genomes project. We thus classify this entry as pathogenic according to the ACMG scoring and inferred classification and as associated to hereditary breast and ovarian cancer syndrome. However, since the gene is autosomal recessive, both alleles need to be affected in order to cause the disorder.

For Daughter we did not find any mutation in the panels OMIM disease genes and ACMG 59 after our filtering criteria. One mutation passes our criteria for the Hereditary Cancer panel, corresponding to the variant located in chr7:116771936 (rs56391007) producing C → T; c.3029C>T; p.Thr1010Ile, located in the *MET* gene, identified for Father as well. As above, we conclude that this variant is of uncertain significance.

We prioritize the filtered variants from Aunt's variant file. After performing the selection of variants above the VCF threshold quality of 20, we identify three variants. Among these three variants, we decided to discard a homozygous variant corresponding to chr1:25563142 (rs121908326) in the *LDLRAP1* gene, as this one did not qualify to have a sufficient genotype quality (minimum acceptable threshold = 40). Among the remaining two variants, the first one corresponds to chr11:111764842 (rs1805076), producing C → T; c.269G>A; p.Gly90Asp in *PPP2R1B*, already observed in Son and Mother. We apply the same criteria as above, inferring this variant effect being of uncertain significance. The remaining heterozygous variant, chr11:108272812 (rs587776549) in the *ATM* gene, produces a frameshift mutation CATC → CTGATc.3245_3247delinsTGAT p.His1082LeufsTer14. The ATM protein plays a critical role assisting cells in recognizing damaged or broken DNA strands, enabling them to repair broken strands and work on maintenance of the stability of the cell's genetic information. The mutation identified here has been described as pathogenic, involved in ataxia-telangiectasia syndrome and has also been linked to a hereditary cancer predisposition (Laake et al., 2000). Mutations to the *ATM* gene have a 20% to 30% lifetime risk of lymphoid, gastric, breast, central nervous system and skin, including melanoma (Choi et al., 2016). We conclude this variant as being pathogenic.

Given the limitations of the VCF file produced for Aunt, for the reminder of the results section, we are only able to perform further analyses for Father, Mother, Daughter and Son; analyses which include genetic risk scores, pharmacogenomics and nutrition/fitness traits.

### Genetic Risk Scores

From our initial list of 49 GWAS phenotypes (Supplementary Table 1), we identified members of the family who have a risk score (or predisposition) of one or two standard deviations (SD) from the average risk score of the 1000 genomes population for the same condition. Table 5 shows the phenotypes whose genetic score from the initial list is more than 2SD (yellow) and those with more than 1SD (green). The GWAS studies from which the genetic risk SNPs originated are sourced in the “Reference Studies” column.

**Table 5:** Phenotypes for family members with <1SD genetic risk score.

**Category**	**Phenotype**	**Father**	**Mother**	**Daughter**	**Son**	**Reference Studies**
General health	Inflammatory bowel disease					Liu et al., 2015
	Ulcerative colitis					Berndt et al., 2013
	Obesity					Berndt et al., 2013
Lipids	Triglycerides					Willer et al., 2013
Cancer	Bladder carcinoma					Kiemeney et al., 2008, 2010; Wu et al., 2009b; Rothman et al., 2010; Rafnar et al., 2011, 2014; Figueroa et al., 2014; Matsuda et al., 2015; Wang et al., 2016
	Breast cancer					Howard et al., 2018
	Glaucoma					Choquet et al., 2018
	Prostate cancer					Schumacher et al., 2018
Mental health	Bipolar disorder					Ferreira et al., 2008; Smith et al., 2009; Cichon et al., 2011; Psychiatric and Consortium Bipolar Disorder Working Group, 2011; Mühleisen et al., 2014; Hou et al., 2016; Ikeda et al., 2018
	Depression					Howard et al., 2018
	Posttraumatic stress disorder					Nievergelt et al., 2015; Stein et al., 2016
	Schizophrenia					Schizophrenia Working Group of the Psychiatric Genomics Consortium, 2014
Dependence /withdrawal	Alcohol dependence					Gelernter et al., 2014; Mbarek et al., 2015
	Nicotine withdrawal					Hällfors et al., 2019
Fitness	Heart rate recovery					Ramírez et al., 2018
Nutrition	Caffeine metabolism					Cornelis et al., 2016
Appearance	Male pattern baldness					Pirastu et al., 2017

*We then calculate their average score and standard deviation (SD). For each family participant we calculate their genetic risk score in the same way as individuals from the 1000 Genomes Project and mark as yellow if his/her risk score is >2SD of the 1000 Genomes Project average and green if his/her risk score is >1SD of the average in 1000 Genomes Project individuals. Both Son and Mother have a >2SD risk score for nicotine withdrawal symptoms. For ulcerative colitis, Mother and Son have a >2SD score (yellow) and Daughter a >1SD score (green). The increased risk of inflammatory bowel disease for both Mother and Daughter overlaps with their predicted ulcerative colitis susceptibility. Father has a >2SD risk of autism spectrum disorder. Autism spectrum disorder genetic risk appears also for the two children (>1SD; Daughter, Son). The alcohol dependence genetic risk score reflects another paternal line predicted predisposition inherited by both children. Since no one in the family to date has been predicted to suffer from autism spectrum disorder or alcohol dependence, it is not possible to confirm this result. A slightly increased risk of obesity in Father and Son is also predicted by this multigenic risk score calculation*.

First, we find that no one phenotype in yellow (>2SD) occurs in isolation. There is either another member of the family in yellow or green (>1SD). This occurs for the predicted higher risks of ulcerative colitis and nicotine withdrawal symptoms. Second, we observe that related phenotypes with high risk overlap (although not always) in the same family individual. For instance, ulcerative colitis is a subtype of inflammatory bowel disease (Ronald et al., 2006; Wu et al., 2009a; Uhlig and Muise, 2017). We find that Mother and Daughter have risks overlapping both phenotypes whereas Son only ulcerative colitis. Father has a risk for three of the four phenotypes for the mental health category. For the disorders where we observe this overlapping phenotype risk, there is scientific literature (Ronald et al., 2006; Wu et al., 2009a; Uhlig and Muise, 2017) supporting this pattern. Third, there are high risk phenotypes in a parent also observed in their offspring. For instance, ulcerative colitis is not present in Father but it is predicted high risk in Mother together with both children displaying inherited risk.

If we describe results according to categories, for general health, we thus find that ulcerative colitis constitutes a phenotype where the risk of the condition is shared among several family members (Mother, Daughter, Son; Supplementary Figures 1A,B). The high risk for ulcerative colitis also overlaps with the higher than average (>1SD) risk of inflammatory bowel disease in two same family members (Mother, Daughter). For cancer there is a more elevated than normal (>1SD) predicted risk of breast cancer among Mother and Daughter, with some isolated moderately high (>1SD) predicted risks of bladder carcinoma for Daughter, glaucoma for Son and prostate cancer for Father. For mental health, Father has higher than normal (>1SD) predicted risk on bipolar disorder, depression, and posttraumatic disorder. Mental health diseases share similar markers thus influencing the greater number of potentially deleterious yet related phenotypes observed in Father. For dependence and withdrawal symptoms phenotypes we find that the paternal line has a higher than average alcohol (>1SD) dependence predicted risk whereas the maternal line passes on to Son a high predicted risk (>2SD) of nicotine withdrawal symptoms. Phenotypes in green (>1SD) that are not shared with other family members are not considered any further. Graphical representation of the ulcerative colitis results using both the whole 1000 Genomes background population (2,504 individuals) and only the Europeans (503) may be found in Supplementary Figure 1.

### Pharmacogenomics

We analyzed the metaboliser status of three cytochrome P450 genes (*CYP2C9, CYP2C19, CYP2D6)* affecting pharmacological responses in Father, Mother, Daughter, and Son. We also look at some pharmacology-related SNPs in additional genes.

*CYP2C9* is responsible for the metabolic clearance of up to 15–20% of all drugs undergoing Phase 1 metabolisation, including warfarin, phenytoin, and oral hypoglycaemics (source: Get to Know an Enzyme: CYP2C9^1^). Some of the more potent *CYP2C9* inhibitors include amiodarone, fluorouracil, metronidazole, and sulphaphenazole. Dangerous drug-drug interaction can arise when an inhibitor is added to a therapeutic regime that includes drugs with a low therapeutic index, such as s-warfarin. Inducers, such as rifampicin, can substantially increase *CYP2C9* activity (source: Get to Know an Enzyme: CYP2C9). For *CYP2C9*, Father, Mother and Son have a predicted metaboliser status of intermediate (^*^1/^*^2). For Daughter, the predicted metaboliser status for *CYP2C9* is poor (^*^2/^*^2).

Warfarin is an anticoagulant used in the prevention and treatment of venous thrombosis, pulmonary embolism, and the complications associated with atrial fibrillation and/or cardiac valve replacement (Dean, 2018). Warfarin metabolism is influenced by genetic polymorphisms in *CYP2C9* and *VKORC1* (Biss et al., 2012). Carriers of the common allelic variants (^*^2 or ^*^3) of the *CYP2C9* are associated with a lower warfarin dose requirement accompanied by a greater tendency to experience haemorrhagic complications. In addition, adults with *VKORC1* (rs9923231) CC alleles require higher warfarin doses than TC or TT. Based on these alleles, we found that Son and Father have a ^*^1/^*^2 *CYP2C9* variant and a TT for rs9923231. Mother has a ^*^1/^*^2 *CYP2C9* variant and a CT for rs9923231. Daughter has a ^*^2/^*^2 *CYP2C9* variant and a TT for rs9923231. This makes Son, Father and Mother intermediate metabolisers and Daughter a poor metaboliser of warfarin.

*CYP2C19* is a liver enzyme that acts on at least 10% of drugs in current clinical use (source: Genetics Home Reference^2^; see references), most notably the antiplatelet treatment clopidogrel (Plavix) but also drugs that treat pain associated with ulcers, such as omeprazole, antiseizure drugs such as mephenytoin, the antimalarial proguanil, and the anxiolytic diazepam. For this gene we found Son, Mother and Daughter to be predicted normal metabolisers (^*^1/^*^1) whereas Father is predicted an intermediate metaboliser (^*^17/^*^4A).

For *CYP2D6*, the final cytochrome we analyse here, we are able to estimate the metabolism and elimination of approximately 25% of clinically used drugs including the opiate codeine (Wang et al., 2009). *CYP2D6* is highly polymorphic in the human population, with marked inter-racial variation observed. Individuals are identified as ultra-rapid (UM), extensive (EM), intermediate (IM) or poor metaboliser (PM), according to the number of functional alleles.

For members of this family we find that there is considerable variation in the alleles detected. For Son and Father, we find them to be predicted extensive metabolisers (^*^2/^*^41 and ^*^1/^*^2, respectively). Mother has the following star alleles ^*^10/^*^2/^*^41/^*^4 [activity score: 0.5–1 (Gaedigk et al., 2018)] which make her predicted range between an intermediate and extensive metaboliser. Daughter has ^*^10/^*^4/^*^20, which makes her a poor or intermediate predicted metaboliser (activity score 0–0.5).

rs12979860 is a SNP near the *IL28B* gene, encoding interferon-lambda-3 (IFN-lambda-3). This SNP influences hepatitis C treatment-induced viral clearance. It is associated with an approximately twofold change in response to pegylated interferon-alpha (PEG-IFN-alpha) plus ribavirin (RBV) treatment, both among patients of European ancestry (*p* = 1.06 x 10e-25). Research indicates that the virus was eradicated in ~80% of CC patients, compared to only about 25% of those with TT, while CT response was intermediate (Elkader and Sproule, 2005). We found that Son and Father carry a CC genotype, whereas Mother and Daughter carry a CT genotype.

### Fitness Trait Analysis

#### Filtering

First, we performed a filtering of the SNPs associated with fitness traits in order to determine which of them should be applied to our family cohort. The full results of our filtering can be found in Table 3. From a total of 10 markers initially selected for genotyping, we classified two as “Convincing” (VO_2_max, and rs1815739 for *ACTN3*), five as “Probable,” two as “Possible” and one “Not demonstrated.” As an example of the application of this framework, in Table 3, the best studied SNP marker is rs1815739 for *ACTN3*. We identified 24 studies for rs1815739 in fitness, most of which suggested a significant decrease in muscle performance by the effect allele (also known as X allele). Based on these studies, we classify the biological plausibility of this marker as high. Our scientific evidence assessment for rs1815739 is “Convincing.”. For “increased performance with caffeine,” we assess existing scientific evidence as “Probable” because despite finding 7 studies, the total number of participants summed by all seven studies is only 250. Within those, there is also one study not showing significant differences in performance with coffee intervention (Pataky et al., 2016; Salinero et al., 2017; Guest et al., 2018; Puente et al., 2018; Carswell et al., 2020; Grgic et al., 2020; Muñoz et al., 2020).

For family trait analysis, we only apply those markers that are either classified as “Convincing” or “Probable”. In the next section we describe in detail our selected fitness analysis results.

#### Fitness Trait Analysis Performed on the Family Cohort

Table 6 summarizes the fitness traits analyzed for 4 family members. Concerning VO_2_Max trainability, training response markers within the 21 SNP panel show Son scoring 13/21 favorable alleles, Father and Mother 16/21 favorable alleles and Daughter scores 15/21 favorable alleles. This contrasts with ≥19 of these alleles associated with elite athletes (Bouchard et al., 2011; Rice et al., 2012; Ghosh et al., 2013; Williams et al., 2017).

**Table 6:** Summary of fitness trait analysis for 4 family members.

**Trait**	**RSID**	**Gene**	**Scientific validity score**	**Father**	**Mother**	**Daughter**	**Son**
VO_2_Max	21	Multiple	Convincing	16/21	16/21	15/21	13/21
Muscle performance	rs1815739	*ACTN3*	Convincing	XR	XR	XR	XX
Caffeine sensitivity/Increased exercise performance with caffeine	rs762551	*CYP1A2*	Probable	AA	CA	AA	AA
Endurance	rs4253778	*PPARA*	Probable	GC	CC	GC	CC
Lactate blood levels	rs1049434	*MCT1*	Probable	TT	TT	TT	TT
Osmotic balance by water support	rs1049305	*AQP1*	Probable	GC	GG	GC	GC
Performance	rs12594956	NRF-2	Probable	CA	CC	CC	CA

*The table shows the observed genotype or (for VO_2_max) favorable alleles for those traits whose scientific evidence assessment was judged as “Convincing” or “Probable”*.

The *ACTN3* R577X (rs1815739) C>T base substitution results in the transformation of an arginine amino acid (R) to a premature stop codon (X). X allele homozygotes are deficient in the alpha-actinin-3 protein, which is associated with a lower fast-twitch fiber percentage and potentially increased injury risk (Yang et al., 2003; Massidda et al., 2019). We found that Father, Mother and Daughter have a CT genotype (XR); whereas Son, harbors a homozygote X allele genotype (XX).

A polymorphism in the *CYP1A2* gene (rs762551; AA genotype) has been associated with improved exercise performance when combined with caffeine intake, with no effect for those with the AC genotype and diminished performance in those with the CC genotype (Guest et al., 2018). We found that most family members (Son, Father, and Daughter) had an AA genotype for this SNP, whereas Mother had a CA genotype.

The role of the peroxisome proliferator activated receptor alpha (*PPARA*) gene intron 7 G/C polymorphism (rs4253778) is also tested in the family. Athletes with high ability in endurance sports have a higher frequency of the G allele (Lopez-Leon et al., 2016). We found that Son and Mother did not have any of the G allele, whereas Father and Daughter had a G allele each.

For the *MCT1* gene's rs1049434, we find all family members to have the TT genotype, associated with lower lactate levels (Cupeiro et al., 2012; Fedotovskaya et al., 2014; Ben-Zaken et al., 2015; Kikuchi et al., 2017a). For the *AQP1* gene, which is associated with osmotic balance and fluid loss when exercising, possession of the C allele has been associated with faster cardiorespiratory endurance (Rivera and Fahey, 2019). For this gene, we found C (favorable) alleles in Son, Father, and Daughter, while no C alleles were found in Mother. Finally, for rs12594956 in *NRF-2*, we find that the genotypes observed (CA/CC) in all family members are not associated with the effect allele (He et al., 2007; Eynon et al., 2010, 2013; Peplonska et al., 2017).

### Nutrition Trait Analysis

Our analysis includes markers involved in the metabolism of main components of diet: carbohydrates, fats, and proteins. We also look at metabolization of essential nutritional components such as vitamins, minerals, and specific dietary substances like lactose, whose metabolism is strongly linked to a genetic marker according to our suggested framework. Table 7 provides a summary of the nutrition markers explained in this section.

**Table 7:** Summary of nutrition trait analysis for the 4 family members.

**Trait**	**RSID**	**Gene**	**Scientific validity score**	**Father**	**Mother**	**Daughter**	**Son**
Homocystine levels	rs1801133	*MTHFR*	Convincing	GA	GA	AA	GG
Vitamin B12 level	rs602662	*FUT2*	Convincing	GA	GG	GA	GG
Vitamin C level	rs33972313	*SLC23A1*	Convincing	CC	CC	CC	CC
Vitamin D Metabolism	rs4588	*GC*	Convincing	GT	GT	GT	GG
Vitamin E level	rs964184	*BUD13 / ZNF259*	Convincing	CC	GG	GC	GC
Greater total body adiposity	rs9939609	*FTO*	Convincing	AA	TT	TA	TA
Saturated fat	rs5082	*APOA2*	Probable	AA	GA	AA	AA
Polyunsaturated Fatty Acids	rs174547	*FADS1*	Convincing	TT	TT	TT	TT
Saturated fat/risk of T2D	rs1137101	*LEPR*	Convincing	AG	AG	GG	AG
Iron Overload /Hemochromatosis	rs1800562	*HFE*	Convincing	GG	GG	GG	GG
Celiac disease	rs2187668	*HLA-DQA1*	Convincing	CC	CC	CC	CC
Lactose persistence	rs4988235	*MCM6-LCT*	Convincing	GG	AA	GA	GA
Alzheimer's	rs429358, rs7412	*APOE*	Convincing	ε3/ε3	ε3/ε3	ε3/ε3	ε3/ε3
Alcohol dependence	rs1229984	*ADH1B*	Convincing	CC	TC	CC	CC

*The table follows the scientific validity score presented in Methods and the observed genotype for the specific trait. We only analyse those traits for which there is a convincing or probable genotype x diet intervention scientific evidence. From among these, here we only show those with a predicted effect, except in the case of Alzheimer's and Coronary Artery Disease*.

#### Filtering

We performed a filtering of the SNPs associated with nutrition traits to select SNPS to be applied to our family cohort. The full results of our filtering can be found in Table 5. From a total of 32 markers initially selected for analysis, we classified 13 as “Convincing,” one as “Probable,” five as “Possible”, and 12 “Not demonstrated.” An example of convincing scientific evidence for nutrition interventions in Table 4 includes *MTHFR* (rs1801133). This SNP is said to affect homocysteine concentrations, which are influenced by dietary folate (Boccia et al., 2008, 2009; Clarke et al., 2011; Liew and Gupta, 2015). A large number of studies (*n* = 70) have been performed to date about this interaction, including randomized trials. We evaluate this interaction as having a high biological plausibility. An example of nutrition marker we classify as possible is *BCO1* (rs6564851). According to our research (Table 4), there are 4 studies with a number of total subjects analyzed of 328 (Yabuta et al., 2016; Moran et al., 2019; Amengual et al., 2020; Graßmann et al., 2020). Our judgement of the underlying knowledge of the biological mechanism involved is medium and there are some cases where a potential intervention may not have the desired effect. We do not include this marker in our subsequent analyses.

Same as in the fitness category, for family trait analysis we only apply those markers that are either classified as “Convincing” or “Probable.” In the next section we describe in detail our selected nutrition trait analysis results.

#### Nutrition Trait Analysis Performed on the Family Cohort

The B vitamins contribute to DNA synthesis and methylation, with homocysteine as a by-product of their metabolism associated with coronary heart disease, stroke, and neurological disease (Tanaka et al., 2009). “A” alleles in the rs1801133 SNP within the *MTHFR* gene have been associated with higher homocysteine levels and reduced folic acid processing (Tanaka et al., 2009). We note that Father and Mother have one A allele whereas Daughter has the two A (“detrimental”) alleles. Son has the two G alleles genotype. Next, the presence of the A allele in rs602662 SNP in *FUT2*, has been associated with higher B12 concentrations (Tanaka et al., 2009). We found the presence of an A allele in Father and Daughter, and no A allele presence in the other individuals. With regards to circulating concentrations of vitamin C (L-ascorbic acid), a variation at rs33972313 (*SLC23A1* gene) has been associated with a reduction in circulating concentrations of L-ascorbic acid (Timpson et al., 2010). None of the family members have the predicted detrimental allele. With regards to vitamin D, rs4588 was genotyped. Son was found homozygous for the major allele (GG) and the rest of the family heterozygous for the minor allele (GT). The effect allele for higher a-tocopherol concentration in plasma (G) is found in both alleles in Mother (GG) and one allele in Son and Daughter (Major et al., 2011, 2012, 2014; Wang and Xu, 2019).

With regards to dietary fat, we analyse a number of SNPs in genes involved in nutrition: *FTO, APOA2, FADS1*, and *LEPR*. With regards to rs9939609 *FTO* variant alleles (homozygous = AA and heterozygous = AT), both Son and Daughter are heterozygous for the risk allele and Father is homozygous for the risk allele. Each additional copy of the rs9939609 A allele has been associated with a BMI increase of a mean of 0.10 *Z*-score units, equivalent to ~0.4 kg/m^2^ (Sonestedt et al., 2009; Tanofsky-Kraff et al., 2009; Zhao et al., 2014b). For the observed genotypes in *APOA2* and *FADS1*, there is no associated effect (Yabuta et al., 2016; Huang et al., 2017; Ching et al., 2019; Moran et al., 2019; Amengual et al., 2020; Graßmann et al., 2020; Wang et al., 2020). For *LEPR*, a study found that rs1137101 AG and GG carriers with a high fat total intake had 3.0 times higher risk of obesity and 4.1 times higher risk of high cholesterol levels than those with a low intake of total fat (Domínguez-Reyes et al., 2015). All family members are carriers of the risk allele (G) of rs1137101.

The HFE protein interacts with other proteins on the cell surface to detect the amount of iron in the body (Katsarou et al., 2019). For rs1800562, a SNP in *HFE*, an A allele was not observed in any of the individuals analyzed here. This A allele causes ~85% of all cases of hemochromatosis (Katsarou et al., 2019). The rs2187668 SNP's CC alleles in all family members have not been associated with Celiac disease (van Heel et al., 2007; Hunt et al., 2008). *MCM6-LCT* regulates lactose persistence. According to a recent study (Mattar et al., 2012), both genotypes of rs4988235 GA and AA were associated with the lactase-persistence phenotype, indicating that the presence of one single lactase-persistence allele in the heterozygous state has a dominant effect, rendering the person a lactose digester, whereas the genotype CC, when the lactase-persistence allele T is absent, is consistent with lactose maldigestion. Father's genotype was found to be CC associated with an increased likelihood of being lactose intolerant.

## Discussion

Our main objective is to provide insight into the current development status of personal genomics, using whole genome sequencing, illustrated by a use case of a family of five. To that end, we provide pathogenicity screening, genetic risk scoring, pharmacogenomics and fitness and nutrition trait analysis of the family. This approach is tailored for the situation where knowledge of the disease and lifestyle history of the family is used to “validate” some of the findings. A main limitation of this approach is the *post-hoc* reasoning that only allows to find *true positive* predictions based on the family observations. In contrast, those risks and phenotypes that are not reflected in the family so far can neither be confirmed nor rejected as it is unclear whether those predictions are “wrong” or whether the conditions have not had their time of onset yet. In addition, there are other limitations stemming from the different methodologies and resources used for analysis and interpretation, which we summarize in Table 8.

**Table 8:** List of known limitations of the methodologies we have performed for our analysis and the countermeasures we have adopted to contain them.

**Methodology**	**Limitations**	**Countermeasures**
Short read whole genome sequencing	• There may be errors in the variant calls • The whole genome is not wholly sequenceable • Structural and copy number variants are challenging to identify	• We performed a quality filter for each variant • Assume regions not sequenced to be gene deserts or unable to provide useful functional annotation • We run a consensus set of algorithms for prediction of copy number regions
Genome screening	• Screened only regions covered by genes and nearby regions • Incomplete, inconsistent annotations • Use of knowledge databases with conflicting results	• Selected those genes curated by OMIM where there are known mutations • Assumed that the vast majority of pathogenic mutations occur within or near coding regions • Employed a third-party protocol to interpret pathogenicity (Fabric Protocol; see Methods) • Inference only by overlapping evidence in OMIM and ClinVar, supplemented by literature search, computational algorithms and allele frequency information from established international datasets (e.g., gnomAD) • Classification of pathogenicity performed by two independent experts
Genetic risk scores	• GWAS only capture highly significant markers, missing less strongly associated markers with the trait • There may be different studies for a trait and there are challenges when integrating them into a single genetic risk score • GWAS is overwhelmingly European • Genetic risk scores may capture only a small amount of genetic risk	• We choose those studies that are of greatest number of participants, preferably from recognizable consortia, to allow the greatest possible number of markers when defining a contribution to susceptibility • We make use of the curation effort of the GWAS Catalog to select studies and markers • We compare GWAS scores with a background population (1000 Genomes Project) and check that our family participants are matched with the same background population when looking for significant differences with the average risk score • We report genetic risks only for patients whose risk is in the extreme tail of risk prediction
Pharmacogenomics	• There is a large amount of variation in pharmacological genes, not all of which can be detected • There may be cases where it is unclear the metaboliser status of a patient • Short read sequencing has limited ability to assess Copy Number Variants and therefore functional duplication or deletions of genes may be missed	• We strictly follow FDA, CPIC, ACMG guidelines when assigning metaboliser status • We make sure that when the metaboliser status is unclear we provide a range of possible eligible options • We run a consensus approach prediction algorithm (Supplementary Materials) to mitigate the risk that we might have failed to detect deletions or duplications within pharmacogenomic genes that may alter their functionality
Fitness	• Small sample sizes; perhaps not so much funding available as for global health conditions • Skewed populations (e.g., mostly European background) • Results often rely on self reporting of adherence to an exercise regimen • Focus in some studies on elite athletes, not necessarily generalisable to the wider population • Traits difficult to phenotype; sensors may only allow indirect measurement (e.g., VO_2_max)	• Adopted an establised framework for trait analysis, so as to exclude studies with a weaker evidentiary basis • Systematically reviewed and assessed the literature choosing only those markers where there is ample evidence of their effect • No inferences of phenotype made based only on fitness marker predictions
Nutrition	• Small sample sizes; perhaps not so much funding available as for global health conditions • Skewed populations (e.g., mostly European background) • Difficult to replicate results; experimental design would use extreme fitness traits (e.g., athletes, which would contribute to difficult replication)	• Adopted an established framework for trait analysis to so as to exclude studies with a weaker evidentiary basis • Systematically reviewed and assessed the literature choosing only those markers where there is ample evidence of their effect • No inferences of phenotype made based only on fitness marker predictions
Validity of inference	• We can only confidently assign true positives	• Performed an in-depth query of the disease and lifestyle history of the family, in order to maximize our ability to confirm positive results • We use overlapping information about family members to explain predictions

For instance, short read whole genome sequencing provides a limited capacity for detecting copy number and structural variants, which are particularly relevant for Pharmacogenomic analysis. To mitigate this shortcoming, we run prediction algorithms (Supplementary Materials) and find no significant prediction of copy number changes in pharmacologically important genes.

For pathogenicity screening, current standards and literature focus on genes (e.g., American College of Medical Genetics and Genomics), and therefore pathogenicity screening does not typically cover intergenic regions. Knowledge bases used for variant annotation may contain inconsistent or incomplete information, and therefore we only report variants where there is consensus among both literature, database and bioinformatic algorithm prediction, within a set of established guidelines. Moreover, while the field of genetics is evolving constantly, it is also a well-known limitation that many variants are currently classified as unknown significance. We do not report variants of unknown significance, but ensure that we use databases that remain current so that we can deploy the latest variant research in the analysis.

Concerning trait analysis in fitness and nutrition, we set a framework for selection and validation of fitness and nutrition markers to mitigate the limitations specific to phenotypes in these areas (smaller study sizes, weaker phenotype – genotype relationships). Application of this framework results in a reduction in the number of markers we were able to test in our family members. Although this filtering has restricted the number of resulting inferences, it has increased the robustness of the analysis.

Finally, the genetic risk analysis we provide here has not been tested in an independent population, and as such serves as an illustration of a potential approach and a template for further work.

### Patterns of Inheritance in Pathogenicity Screening

When screening for pathogenicity we find that Son and Father have C → T; c.200C>T; p.Thr67Ile within the *CTH* gene. Father and Daughter share the mutation C → T; c.3029C>T; p.Thr1010Ile, located in the *MET* gene. Son and Mother share C → T; c.269G>A; p.Gly90Asp in *PPP2R1B*. All of these mutations are not deemed reportable due to the unknown significance nature of the inferences. The reportable variant is the A → T; c.4912A>T; p.Lys1638Ter in the *BRCA2* gene for Mother in a recessive context. Mother had a benign breast tumor removed in her forties but it was never analyzed. Therefore, it is not possible to ascertain whether her *BRCA2* gene mutation had any role in her benign tumor formation. Fortunately, this mutation is heterozygous and Father does not carry a known pathogenic mutation in this gene. Both children did not inherit Mother's pathogenic *BRCA2* mutation and therefore are unable to pass it on to their offspring.

Aunt passed away in 2013, aged 79, due to a metastasised melanoma. For this participant, we transform our screening into a quasi-diagnostic setting given that we would like to identify a potential genetic cause for her demise. We were able to retrieve 36 hairs 4 years after her death from one of her combs. The DNA was carefully handled (see Supplementary Materials). We were able to assess pathogenicity among those variants that passed our strict quality filters. A heterozygous frameshift mutation, chr11:108272812 (rs587776549) in the *ATM* gene was identified. Recently, the Pan-Cancer Analysis of Whole Genomes Consortium confirmed that many cancer driver mutations are two-hit inactivation events (ICGC/TCGA Pan-Cancer Analysis of Whole Genomes Consortium, 2020), with 17% of patients having rare germline protein-truncating variants (PTVs) in cancer-predisposition genes, DNA-damage response genes and somatic driver genes. Biallelic inactivation due to somatic alteration on top of a germline PTV was observed in 4.5% of patients overall, with 81% of these affecting known cancer-predisposition genes (such as *ATM*). We thus hypothesize that the loss of function of one copy of the *ATM* gene could have contributed to her melanoma. Although Aunt's genome only provides information about her germline genetics and not the actual somatic mutations that led to the disease that ended her life, a more targeted cancer therapy (than the general chemotherapy she was administrated with) targeting defects in the DNA repair caused by *ATM* was already available while she was still alive (Kelley et al., 2014) and was never used.

### Genetic Risk Scores

We observe there is a conserved family risk of ulcerative colitis, running in Son, Mother, and Daughter. Ulcerative colitis is a long-term condition that results in inflammation and ulcers of the colon and rectum. It has also been found that both Mother and Daughter have a >1SD risk of inflammatory bowel disease, of which ulcerative colitis is a type. The primary symptoms of active disease are abdominal pain and diarrhea mixed with blood. Mother has reported suffering from a recurrent abdominal pain associated with inflammation of her colon. Her symptoms have appeared intermittently but are more recurrent in older age, affecting her quality of life. Given that ulcerative colitis begins most commonly between the ages of 15 and 25 with a second peak of onset in the 6th decade of life, Mother's reported symptoms are concordant with her ulcerative colitis / bowel disease susceptibility. We also note that according to Sen and Stark (2019), CYP2D6^*^4 polymorphisms may be risk factors for ulcerative colitis. Both Mother and Daughter display CYP2D6^*^4.

### Pharmacological Management

We have noted that for warfarin, the genotyping analysis has shown that members of the family are either intermediate or poor metabolisers. According to FDA guidance (Dean, 2012), Daughter requires 20% of the standard initial recommended dose and would take a more prolonged time to achieve the maximum anticoagulant effect. Son, Father, and Mother require a 60% of the standard initial recommended dose. This information is particularly relevant to Father, who was recently diagnosed with atrial fibrillation. Atrial fibrillation is a heart condition that causes an irregular and often abnormally fast heart rate. People with atrial fibrillation who have a high or moderate risk of having a stroke are usually prescribed warfarin. This was the case of Father, who was recommended to take warfarin to stop the risk of blood clotting. It has been reported by Father, that as soon as he started taking warfarin, he began to experience sores in legs, changes in the skin color, and severe pain in his lower half of the body. We note that his predicted response to warfarin is concordant with warfarin sensitivity (Vu and Gooderham, 2017). Hence, knowledge of this genetic predisposition would have been helpful to the clinician when making an initial prescription.

We also note that for both Mother and Daughter their predicted metaboliser status for *CYP2D6* is either intermediate or poor. This has important implications in the specific dosage required by these individuals to receive the appropriate effects for pain relievers such as codeine and tramadol (Smith et al., 2019). So far there is some anecdotal evidence that Mother and Daughter are not able to cope well opiates, but nothing that was confirmed medically. Of note, the three most susceptible individuals to ulcerative colitis, Mother, Son, and Daughter are predicted to be normal metabolisers of drugs that treat pain associated with ulcers, such as omeprazole (Dickinson, 1994).

### Fitness

We performed an investigation of the literature to identify candidate fitness gene x interactions and their relation to a health outcome (see Methods section). Several limitations were noted throughout these studies, including the robustness of significance for identified variants, small sample sizes, limited cohorts focused primarily on Caucasian populations, and minimal baseline data (Williams et al., 2017). These factors are combined with differences in exercise training programs, diet and other environmental gene expression mediators between studies. As a result, we are able to classify as “Convincing” (2 of 10 candidates) or “Probable” (5 of 10). Overall, we found that fitness studies were made with a smaller sample size compared to nutrition. For instance, *ACTN3*'s rs1815739, one of the most studied fitness-related SNPs, there are >1000 participants studied in total, which would put this SNP among the lowest sample sizes if included in nutrition markers, where we found seven markers with >100,000 study participants.

For all family members, the predicted genetic VO_2_max trainability was predicted average or less than average, contrasting with their lower predicted levels of blood lactate accumulation. With the exception of Mother, the family harbor variants in *AQP1* alleles associated with endurance and fluid balance. Their genotype also predicts a predisposition to improved exercise performance if done with caffeine [with the exception of Mother; (Guest et al., 2018)]. Son is unique in the family in having the XX genotype for rs1815739 in *ACTN3*. Deficiency in α-actinin-3 can be accompanied by higher body fatness, lower muscle strength and higher muscle flexibility and range of motion (Yang et al., 2003; Massidda et al., 2019). A study suggested that recreational marathon runners who have the ACTN3 XX genotype could benefit from personalized strength training to improve their performance more than their counterparts with other *ACTN3* genotypes (Del Coso et al., 2019b).

### Nutrition

Compared to fitness studies, we found a greater number of candidate nutrition phenotypes passed our filtering (14 out of 32 initially selected phenotypes; Table 5). Larger sample sizes and a greater number of studies with concordant results were the main reasons for a larger number of nutrition phenotypes passing our filtering. As with fitness, we choose to analyse those traits whose scientific validity score is convincing or probable and report those that are likely to display pointers for further action or deemed reportable given the family disease and lifestyle history.

Congruent with the general lower likelihood of predicted alcohol dependence by rs1229984 in *ADH1B*, there is no history of alcohol addiction in the family. For all members of the family except Mother, there is a predicted increase in total body adiposity as suggested by *FTO* rs9939609. With regards to vitamin-related traits, all family members with the exception of Son are predicted to be less likely to respond to vitamin D supplements. For vitamin B12 levels, Mother and Son are predicted to harbor lower B12 levels and higher for Father and Daughter. All family members are predicted lower serum L-ascorbic acid.

For homocysteine levels, we found that all family members except Son are predicted to be higher. Reduction of plasma homocysteine levels has been observed with supplementation of vitamin B12 and folic acid (Boccia et al., 2008; Clarke et al., 2011; Liew and Gupta, 2015). Several other studies have observed associations between lower circulating vitamin B12 levels and adverse metabolic health profiles, with insulin resistance, cardiovascular disorders, and adiposity as important features (Hazra et al., 2009; Tanwar et al., 2013; Allin et al., 2017; Nongmaithem et al., 2017; Zhao and Schooling, 2017).

With regards to lactose consumption, we were able to confirm that Father, suspected to be lactose intolerant, has the lactose intolerant genotype.

### Negative Findings

When performing a screening study in an individual, for some variants which can confer significant disease risk, it is important to report not just positive findings, but also negative ones if there is family history of the disease. The ApoE2, E3, and E4 isoforms, which are encoded by the ε2, ε3, and ε4 alleles of the *APOE* gene, respectively, differ from one another at amino acid residues 112 and/or 158. There is a significant association between the ε4 allele of *APOE* and Alzheimer's disease. *APOE* ε4 increases the risk of Alzheimer's disease and lowers the age of disease onset in a gene-dose-dependent manner (Liu et al., 2013). A small proportion of apo ε2 homozygotes, develop type III hyperlipoproteinemia, a highly atherogenic form disorder of lipoprotein metabolism characterized by the accumulation of remnant particles derived from the incomplete catabolism of triglyceride-rich lipoproteins (März et al., 2000). All the family members whose genomes were analyzed for this study exhibit the wild type ε3/ε3, meaning that no association to Alzheimer's disease is conferred. This is also further supported by analysis we performed for family members using genetic risks of Alzheimer's disease (Supplementary Table 1). The fact that there is history of Alzheimer's disease in the maternal line, makes it interesting to ascertain whether genetic risk for this disease is present in the family. It was thus of special interest for the family to research this trait, with the positive outcome that all family members display the less risky ε3/ε3 alleles. We were also in search of negative findings for classified pathogenic mutations that fall within any of the ACMG 59 genes and were able to find only one positive finding for Mother in *BRCA2*. Our variant analysis did not find any other mutation within the 59 genes. As always, the fact that no mutation was found does not necessarily mean a particular disease might not develop.

### Integration of Results

Part of the novelty of the present study revolves around the integration of genetic screening, genetic risk scores and trait analysis. A further layer of integration is constituted by the familial context our participant dataset provides. As stated in Methods, each family individual is tested independently for each of the genetic screening panels, genetic score phenotypes and trait analysis. Although the overlap between each of these methods can only be partial, we now explore the degree of consistency and support that each of the results conveys.

For obesity, the family history indicates a persistent tendency toward this phenotype. At the level of genotyping, the rs9939609 *FTO* marker analysis, shown to be the most contributing to obesity (Sonestedt et al., 2009; Tanofsky-Kraff et al., 2009; Zhao et al., 2014b), yields all genotyped individuals except Mother to carry the risk allele. We acknowledge that the specific contribution of this SNP can only be small. When integrating this genotypic result with GWAS-based genetic risk score, we observe that the obesity risk slightly increased (>1SD) for Father and Son.

Both analysis of specific SNPs in the APOE gene (see Table 7), and genetic risk scores do not suggest an increased risk for all family members of Alzheimer's disease. This is also congruent with the observed disease history of the analyzed family, where both parents are highly advanced in years of age (mid-eighties) and no signs for the disease have been observed yet. This does not rule out the possibility that any member of the family could develop Alzheimer's disease at any point in the future. It does rule out, however, both parents having developed early onset Alzheimer's disease. For alcohol dependency, there has not been observed any tendency of addictive behavior in the family. The rs1229984 *ADH1B* marker supports this phenotype. However, the >1SD predicted genetic risk for alcohol dependency in Father, Daughter and Son does not. A way to reconciling this result is that the genetic risk is moderately higher than average and therefore it does not strongly rule out the possibility of a false positive or random fluctuation, since the observed genetic risk for alcohol dependence may be the result of fluctuations in the score that are not significant. Another integration of different analysis sources is the pathogenic heterozygous variant for *BRCA2* p.Lys1638Ter observed in Mother and her >1SD genetic risk observed for breast cancer. An interpretation of this finding is that she only carries one defective allele for the gene, increasing her risk but not high enough to make it to our >2SD average score threshold.

With regards to integrating the results of similar (yet independent) tests performed in different individuals of the family, we note the coincidence of phenotypic history of irritable colon of Mother with her >2SD increased risk of ulcerative colitis. As mentioned earlier, this >2SD phenotype risk is also observed in Son and not so strongly in Daughter (>1SD), suggesting a pointer for preventative action on the part of Son.

### Communication and Attitudes Regarding Actionable Results

Results for members of the family were communicated either in person or via phone call. For Son, his results have had an impact in his training exercise program, which has a lot more stretching and warming up, with less emphasis on speed and more on building up his endurance. The predicted ulcerative colitis risk for three members of the family was communicated to Mother, who is already displaying some symptoms of the disease, and to Son, who is already taking steps to bring these results forward to his general practitioner as part of his future health management plans. Father's possible explanation of his adverse reaction to warfarin has also been discussed and he has currently discontinued taking the medicine, having discussed it first with his cardiologist. The communication of Aunt's result of her mutation in the *ATM* gene has not led to any concrete actions by her partner.

The family has been exposed to genetic testing for a decade (Corpas, 2012), and as such were generally comfortable knowing results of genetic analysis. Even so, attention was paid in particular to make sure they were aware of the ramifications of knowing their results of tests related to more serious and harder to treat conditions (such as ulcerative colitis).

As was the case when the same family was analyzed with direct-to-consumer genotyping methods (Corpas, 2012), the tendency to discuss “whose genome is best” is a recurrent pattern that could affect other families when communicating genetic test results. We stress the importance of discussing such results with qualified professionals such as genetic counselors.

Compared to the communication of the results in 2012, we also note the change in attitude toward sharing of personal genomic data. Individuals are less keen to share their genetic data now, arguing that their perceptions regarding the privacy of their data have been changed by their increased awareness of the importance of protecting individual's personal data.

## Conclusion

By looking at the genome from various methodological angles and applying distinct analytical frameworks as appropriate, we were able to build a “genetic story” of each individual. We built this story in part through having whole genomes as the basis for the analysis. The approach is applied here for a family, but we believe it is also valid for individuals. Our most notable findings for the family were around susceptibility to ulcerative colitis, and in the areas of fitness, nutrition, and pharmacogenomics.

Concerning ulcerative colitis, when analyzing genetic risk scores, we noted that the recurrent intestinal pain Mother has been affected from for years is concordant with her substantially increased risk of suffering from ulcerative colitis. Moreover, this high risk is predicted in three out of the five members of the family, two of them overlapping with increased risk of inflammatory bowel disease, ulcerative colitis being one type of this disease. We report this susceptibility to ulcerative colitis/inflammatory bowel disease as a potential lead for preventative intervention in at least one family member (Son) who is currently asymptomatic.

We observed some associations for fitness and nutrition variants which passed our quality control framework and as such we believe are valuable for relevant nutritional and exercise science specialists to help the family in making plans in those areas.

We were also able to hypothesize a genetic contribution to the development of melanoma leading to the passing of Aunt. A pathogenic heterozygous germline mutation was reported in her *ATM* gene. This gene has been described as being involved in DNA repair and the information gathered here could have been exploited for targeted cancer therapy if caught on time.

Concordance between an adverse reaction to warfarin and a prediction for low dosing requirement was observed in Father, which he has already acted on, in consultation with his cardiologist. There were also informative results for Mother and Daughter regarding their likely metaboliser status for certain drugs. While not relevant to them at the moment, this information could be shared with their physician in the event that these drugs become necessary in the future, with the hope of reducing trial and error in prescribing and so cutting down the possibility of adverse reactions.

We believe that, taken together, these results represent relevant information which the family can use, when working with the appropriate healthcare professionals, to proactively promote their health and well-being. Any one element of the analysis would not allow this genetic “story” to be compellingly told, but when all them are put together, the narrative becomes more actionable, increasing the applicability of whole genome screening to pre-emptive healthcare and well-being management.

## Data Availability Statement

The sources of genetic markers used in this study are included in Table 3 and Table 4. The sources for genetic risk score creation are included in Supplementary Table 1. The gene panel utilised for cancer screening is in the Supplementary materials. The family genome variation data for this manuscript is not publicly available because they were not consented for open access. Request to access the family genome variation data should be directed to Manuel Corpas (m.corpas@cpm.onl).

## Ethics Statement

We confirm that written informed consent was obtained from the individuals or appropriate next to kin individual for the publication of any potentially identifiable images or data included in this article.

## Author Contributions

MC and EL conceived the experiments and performed the analysis. MC wrote the paper with contributions from all authors. All authors read and approved the paper.

## Conflict of Interest

At the time of writing, MC, KM, AM, and EL are associated with Cambridge Precision Medicine Limited. VM is a full-time employee of Fabric Genomics.

## Footnotes

^1^Get to Know an Enzyme: CYP2C9 Pharmacy Times. Available at: https://www.pharmacytimes.com/publications/issue/2008/2008-03/2008-03-8462 [accessed October 15, 2019].

^2^Genetics Home Reference CYP2C19 gene. Genetics Home Reference. Available at: https://ghr.nlm.nih.gov/gene/CYP2C19 [accessed October 15, 2019].
