# Supplementary material for: Whole Genome Interpretation for a Family of Five
Source: Front Genet. 2021 Mar 8;12:535123. doi: 10.3389/fgene.2021.535123 (PMC7982663; doi:10.3389/fgene.2021.535123)
Supplement: Supplementary file 1 [file Table_1.docx]

Supplementary Materials

## DNA sample preparation and sequencing

Four saliva samples from a family of Iberian Spanish/European descent were collected using oragene OG-600 and sent for DNA extraction and sequencing. The DNA sample was randomly fragmented by Covaris technology and fragments of 350 bps were obtained. Fragment DNA ends were repaired and an ‘A’ base added at the 3’ end of each strand. Adapters were then ligated to both strands of the end repaired/dA tailed DNA fragment. Amplification by ligation-mediated PCR was performed and then single strand separation and cyclisation. DNA nanoballs were created and loaded into the patterned nanoarrays and pair-end reads read through on the BGISEQ-500 platform and high throughput for each library to maximise the chances of a target of 30x coverage. Software for base calling with default parameters and the sequence data of each individual were generated as paired-end reads, identified as ‘raw data’ and provided as FASTQ format.

Our analysis included the genome of a deceased relative whose DNA was extracted four years after her death. We were able to collect a DNA sample from hair follicles in a retrieved comb. For this individual (Aunt, PT00010A), we performed in parallel a set of different sequencing experiments given the level of degradation of the DNA detected as well as the nature of the sample. Hair roots were identified and verified using an inverted microscope. Seven 0.5 – 1 cm pieces were cut off starting from the hair bulb and transferred to a 1.5 ml microcentrifuge tube. DNA extraction was carried out using the QIAamp DNA Investigator Kit from Qiagen, following manufacturer’s instructions. Extracted DNA was quantified using NanoVue^TM^ plus. 2 μl were used as input in the quantification. The DNA concentration measured was 0.452 ng/μl. The sample was then fragmented and its DNA library was prepared and sequenced on the Illumina HiSeq with a sequencing configuration of 2x150 PE. After sequencing was completed, base calls and quality scores were stored in bcl format files. Using bcl2fastq v. 2.17, bcl files were converted into fastq files and de-multiplexed according to the sample’s barcode. Data amount and quality were examined and adapter sequences were removed during the process before the analysis.

## Data Processing

Once fastq files were obtained, we used the Sentieon DNASeq pipeline (Kendig et al., 2019) for all five samples. Sentieon is a toolkit analogous to GATK (McKenna et al., 2010) but built on a highly optimised backend. It takes raw fastq files and maps them to GRCh38 human reference genome using BWA-MEM (Li and Durbin, 2009). For variant calling, Sentieon uses the recommended best practices for variant analysis with GATK (Website), with local realignment around indels and base recalibration using GATK and duplicate reads removed by Picard tools (Picard). The sequencing depth and coverage for each individual were calculated with strict data quality controls throughout the pipeline.

## Prediction of Structural Variants

Structural variants are large variations of the genome (> 50 base pairs). They are difficult to detect directly in short-read data, requiring the utilisation of several types of indirect signals. For their inference, we used Parliament2, a structural variant caller which combines multiple best-in-class structural variant callers to create a highly accurate callset (Zarate et al., 2020). Parliament2 has been deployed as a docker container in the DNAnexus platform (https://platform.dnanexus.com/), allowing us to query the BAM files created from the Sentieon pipeline as input and providing a combined genotype VCF file that contains structural variant predictions from all the algorithms that Parliament2 uses. For Father, Mother, Daughter and Son, we queried their Parliament2 output in search for duplications or deletions within the GRCh38 coordinates of the Cytochrome P450 genes of our pharmacogenomics analysis:

- *CYP2D6*: chr22:42,126,499-42,130,810

- *CYP2C9*: chr10:94,938,658-94,990,091

- *CYP2C19*: chr10:94,762,681-94,855,547

Based on the Parliament2 output, there is not enough support to warrant there are copy number changes in any of these regions (data available via Data Access Agreement with all other inputs and outputs from this analysis).

## Genetic Risk Scores

We present a list of all of the phenotypes for which we have developed a genetic risk score (Supplementary Table 1). Each genetic risk score is based on one or several publications referenced from the GWAS Catalogue. In order to select a study for our phenotypes we performed a curation of candidate studies and selected those that belonged to established Genome Wide Association (GWAS) consortia. Their SNPs would need to include at least their risk allele and odds ratio or beta scores of their weight contribution to the phenotype. We analysed the four saliva sample individuals for all of the genetic risk scores for each of the phenotypes. In this paper we only report those for which there is an individual score greater than one standard deviation from the mean of the background distribution. We group phenotypes by categories.

**Supplementary Table 1**. List of phenotypes for which genetic risk scores were created and tested among the four saliva sample individuals of the quintuplet.

| **Category** | **Phenotype** | **GWAS Catalogue Publication ID** |
| --- | --- | --- |
| General Health | Age related macular degeneration | GCST000652, GCST000653, GCST000806, GCST001100, GCST001571, GCST001814, GCST001884 |
|  | Body mass index | GCST002783 |
|  | Crohn’s disease | GCST003044 |
|  | Glaucoma | GCST006065, GCST006066, GCST006067 |
|  | Hip circumference | GCST004066, GCST004067 |
|  | Inflammatory bowel disease | GCST003043 |
|  | Obesity | GCST2081 |
|  | Rheumatoid arthritis | GCST002318 |
|  | Ulcerative colitis | GCST003045 |
| Heart and lungs | Coronary artery disease | GCST003116 |
|  | Chronic obstructive pulmonary disease | GCST004147 |
|  | Diastolic blood pressure | GCST006020, GCST006021, GCST006022, GCST006023 |
|  | Hypertension | GCST006020, GCST006021, GCST006022, GCST006023 |
|  | Myocardial infarction | GCST003116 |
|  | Pulse pressure | GCST006020, GCST006021, GCST006022, GCST006023 |
|  | Systolic blood pressure | GCST006020, GCST006021, GCST006022, GCST006023 |
| Endocrine | Diabetes type1 | GCST005536 |
|  | Diabetes type2 | GCST002352, GCST005047 |
|  | Hypothyroidism | GCST001474 |
| Lipids | HDL cholesterol | GCST005902 |
|  | LDL cholesterol | GCST005902 |
|  | Total cholesterol | GCST005902 |
|  | Triglycerides | GCST005902 |
| Cancer | Bladder carcinoma | GCST000231, GCST000454, GCST000639, GCST000842, GCST001153, GCST002240, GCST002243, GCST002460, GCST002645, GCST003384 |
|  | Breast cancer | GCST004988 |
|  | Chronic lymphocytic leukemia | GCST000224, GCST000906, GCST001570, GCST002299, GCST004146 |
|  | Lung carcinoma | GCST004748 |
|  | Melanoma | GCST004142 |
|  | Pancreatic cancer | GCST002991, GCST003758, GCST005434, GCST002553 |
|  | Prostate cancer | GCST006085 |
| Neurological | Alzheimer’s disease | GCST003427 |
|  | Migraine disorder | GCST002078, GCST002079, GCST002080, GCST002081 |
|  | Parkinson’s disease | GCST004902 |
|  | Stroke | GCST005842 |
| Mental Health | Autism Spectrum Disorder | GCST004521 |
|  | Bipolar disorder | GCST000220, GCST000417, GCST000985, GCST001241, GCST002385, GCST003724, GCST004139 |
|  | Depression broad | GCST005902 |
|  | Major depressive disorder | GCST005231 |
|  | Posttraumatic stress disorder | GCST002681, GCST003495 |
|  | Schizophrenia | GCST002539 |
| Dependence and withdrawal | Alcohol dependence | GCST003121, GCST004712 |
|  | Nicotine dependence | GCST006300, GCST006301, GCST006302, GCST006303, GCST006869 |
|  | Nicotine withdrawal | GCST006300, GCST006301, GCST006302, GCST006303, GCST006869 |
| Fitness | Heart rate recovery | GCST005788 |
|  | Heart rate response | GCST005787 |
| Nutrition and metabolism | Caffeine metabolism | GCST003851 |
|  | Celiac disease | GCST000157, GCST000612, GCST005523 |
|  | Vitamin D levels | GCST004726 |
| Appearance | Male pattern baldness | GCST005116 |

**Supplementary Figure 1A, 1B, 1C, 1D**: Genetic risk scores for ulcerative colitis for Father (1A), Mother (1B), Daughter (1C) and Son (1D). We show two histograms for each individual, reflecting a difference in the background population. The graphs marked ‘all ethnicities’ are created including a background population of all ethnicities in the 1000 genomes project, comprising 2504 individuals from, Africa (AFR), Ad Mixed America (AMR), East Asia (EAS), Europe (EUR) and South Asian (SAS). In order to control for the possibility that ethnic difference might influence the results, we also ran the scores for each family member with a background population of only the 669 Europeans (EUR) in the 1000 genomes project, the family being of European ancestry. The Y axis of each graph shows the number of individuals who fall within each bin on the X axis. We find that Son and Mother have a >2 standard deviation from the mean score of the background population and that Daughter has a greater than 1 standard deviation from the mean. We find that including only Europeans in the background population does not significantly affect the results. Note that we do not show an absolute score on the graphs, as we are solving for the position of the individual within the background population for the combined effect of each of the 23 SNPs that were curated for this phenotype.


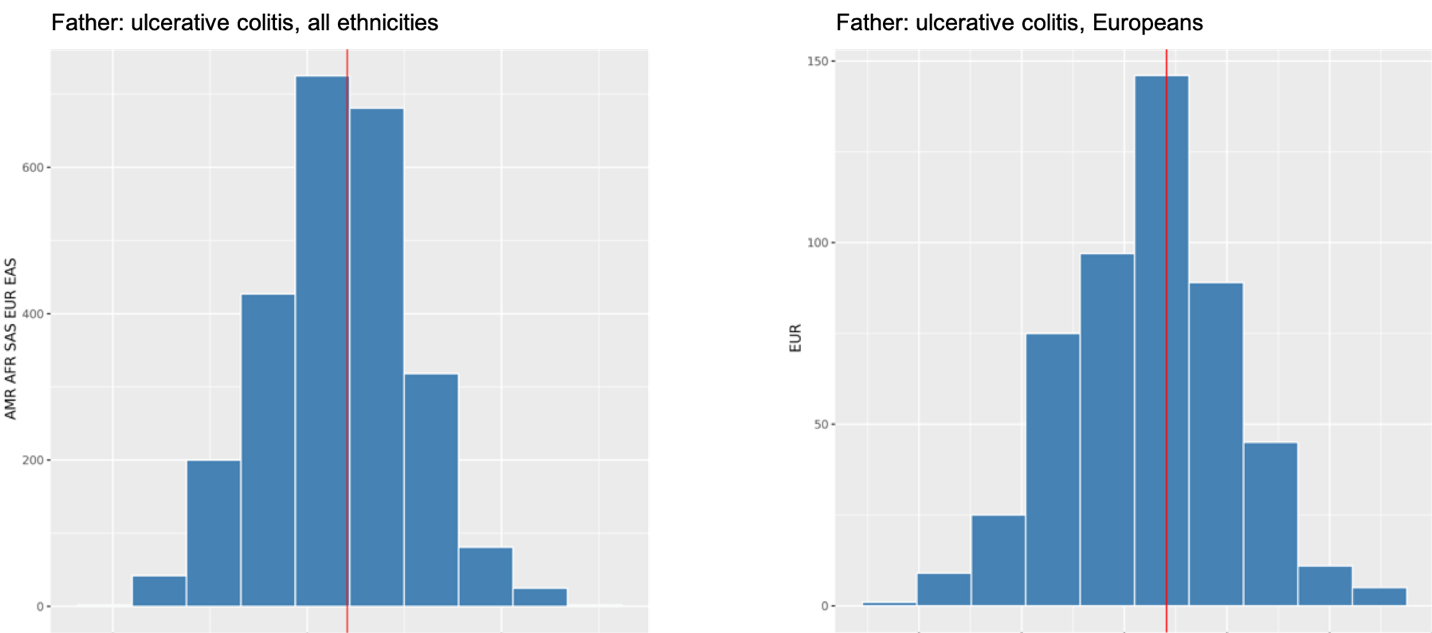


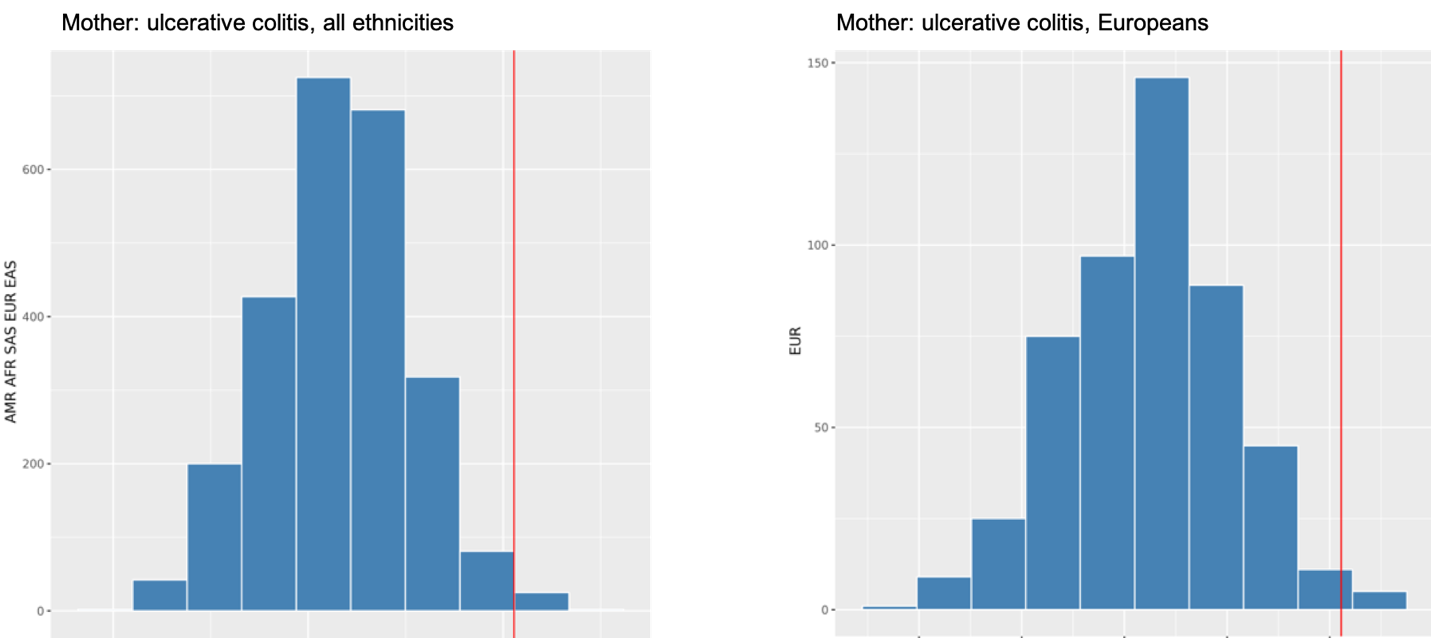


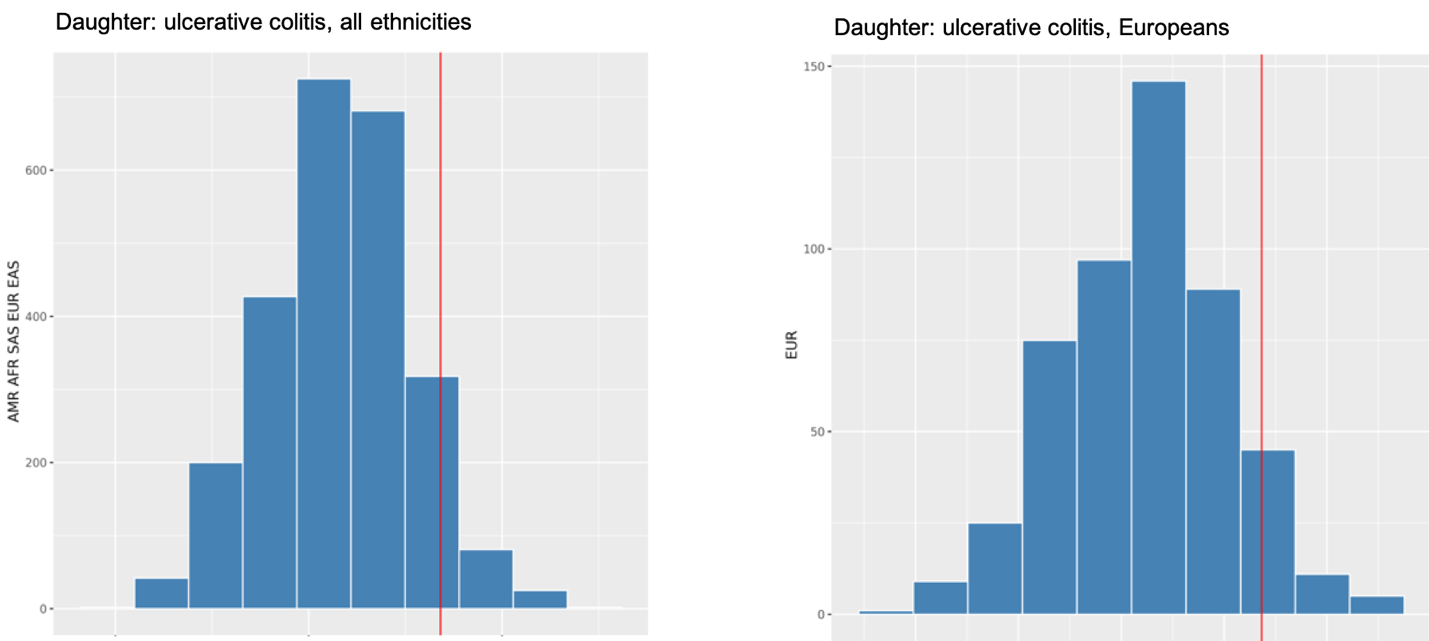


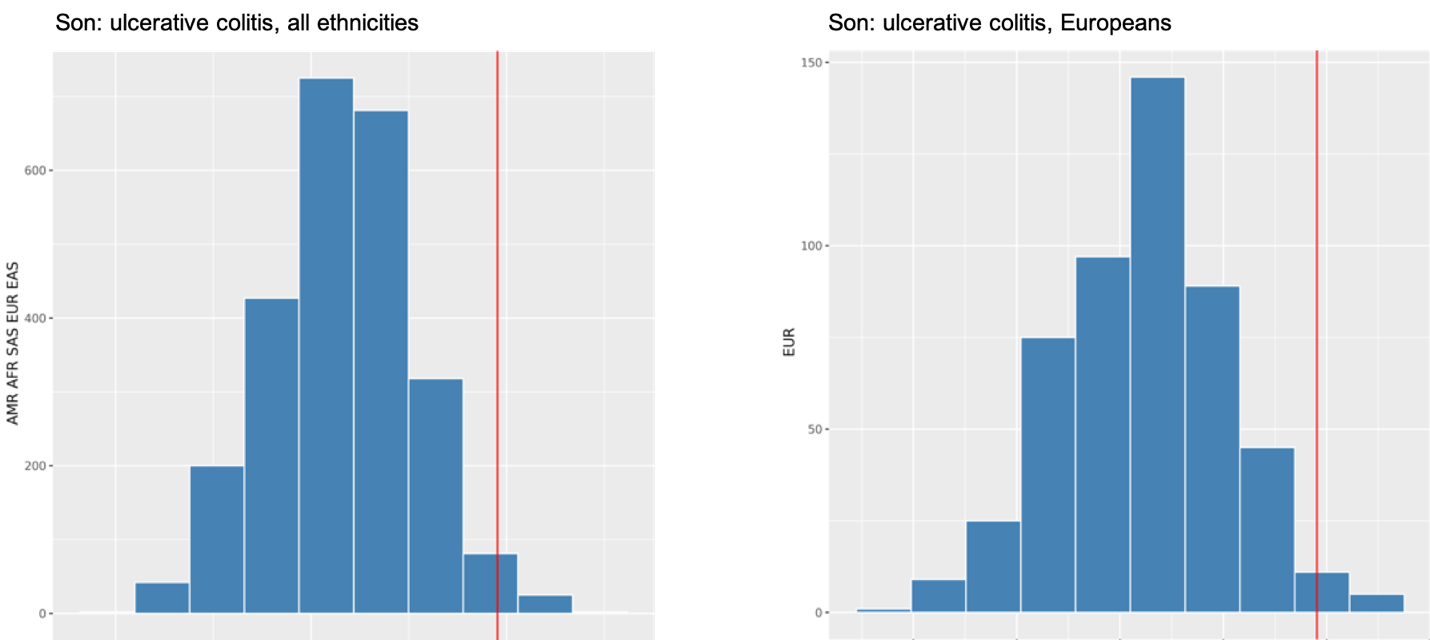


## Hereditary Cancer Gene Panel

This set of genes were chosen because the associated disease condition had been curated by Fabric Genomics (Fabric Hereditary Panels with ACE). These genes require to be specifically curated against the related condition; e.g., for BRCA1, the curated condition is hereditary breast and ovarian cancer. Below is the list of genes we used, separated by commas and no spaces.

ADI1,ALK,APC,ATM,BAP1,BARD1,BLM,BMPR1A,BRCA1,BRCA2,BRIP1,CDH1,CDK4,CDKN2A,CHEK2,DICER1,EPCAM,FANCC,FH,FLCN,HOXB13,MEN1,MET,MLH1,MRE11A,MSH2,MSH6,MUTYH,NBN,NF1,PALB2,PHOX2B,PMS2,POLD1,POLE,POT1,PRSS1,PTCH1,PTEN,RAD50,RAD51C,RAD51D,SDHA,SLX4,SMAD4,SMARCA4,SMARCB1,STK11,SUFU,TP53,XRCC2,XRCC3

References

Fabric Hereditary Panels with ACE Available at: <https://info.fabricgenomics.com/ace> [Accessed January 31, 2021].

Kendig, K. I., Baheti, S., Bockol, M. A., Drucker, T. M., Hart, S. N., Heldenbrand, J. R., et al. (2019). Sentieon DNASeq Variant Calling Workflow Demonstrates Strong Computational Performance and Accuracy. *Front. Genet.* 10, 736.

Li, H., and Durbin, R. (2009). Fast and accurate short read alignment with Burrows-Wheeler transform. *Bioinformatics* 25, 1754–1760.

McKenna, A., Hanna, M., Banks, E., Sivachenko, A., Cibulskis, K., Kernytsky, A., et al. (2010). The Genome Analysis Toolkit: a MapReduce framework for analyzing next-generation DNA sequencing data. *Genome Res.* 20, 1297–1303.

Picard Available at: <http://broadinstitute.github.io/picard/> [Accessed January 31, 2021].

Website Available at: <https://www.broadinstitute.org/gatk/guide/best-practices> [Accessed January 31, 2021].

Zarate, S., Carroll, A., Mahmoud, M., Krasheninina, O., Jun, G., Salerno, W. J., et al. (2020). Parliament2: Accurate structural variant calling at scale. *Gigascience* 9. doi: 10.1093/gigascience/giaa145
